# Application of Dominant Gut Microbiota Promises to Replace Fecal Microbiota Transplantation as a New Treatment for Alzheimer’s Disease

**DOI:** 10.3390/microorganisms11122854

**Published:** 2023-11-24

**Authors:** Mufan Li, Huan Yang, Chenyi Shao, Yinhui Liu, Shu Wen, Li Tang

**Affiliations:** Department of Microecology, College of Basic Medical Sciences, Dalian Medical University, Dalian 116044, China; limufan0722@163.com (M.L.); yang_19962020@163.com (H.Y.); shaocy@dmu.edu.cn (C.S.); yhliu_dl@163.com (Y.L.); shuwen@dmu.edu.cn (S.W.)

**Keywords:** Alzheimer’s disease, fecal microbiota transplantation, brain–gut axis, intestinal dominant flora, probiotics

## Abstract

Several studies have confirmed that the pathophysiological progression of Alzheimer’s disease (AD) is closely related to changes in the intestinal microbiota; thus, modifying the intestinal microbiota has emerged as a new way to treat AD. Effective interventions for gut microbiota include the application of probiotics and other measures such as fecal microbiota transplantation (FMT). However, the application of probiotics ignores that the intestine is a complete microecosystem with competition among microorganisms. FMT also has issues when applied to patient treatment. In a previous study, we found that eight species of bacteria that are isolated with high frequency in the normal intestinal microbiota (i.e., intestinal dominant microbiota) have biological activities consistent with the effects of FMT. In this article, we confirmed that the treatment of intestinal dominant microbiota significantly restored intestinal microbiota abundance and composition to normal levels in APP/PS1 mice; downregulated brain tissue pro-inflammatory cytokines (IL-1β and IL-6) and amyloid precursor protein (APP) and β-site APP cleavage enzyme 1 (BACE1) expression levels; and reduced the area of Aβ plaque deposition in the brain hippocampus. Our study provides a new therapeutic concept for the treatment of AD, adjusting the intestinal microecological balance through dominant intestinal microbiota may be an alternative to FMT.

## 1. Introduction

Alzheimer’s disease (AD) is the most common neurodegenerative disease, affecting more than 50 million people worldwide [1,2]. Neuropathological changes in AD mainly include pathological deposition of extraneuronal β-amyloid (Aβ) peptides, neurofibrillary tangles of hyperphosphorylated neuronal tau proteins, neurotrophic dystrophy, loss of junctional neurons, synaptic loss, and other pathological phenomena [3,4]. This condition is accompanied by the proliferation of immunologically active cells of the central nervous system (astrocytes) as well as the activation of microglia, which places the brain in an inflammatory state [5,6]. However, the pathogenesis of AD is still unclear and the causes of the various pathological phenomena that accompany it cannot be explained [7]. It appears that changes in the intestinal microbiota play a very crucial role in the pathophysiological alterations of AD [8,9,10], and treatment of AD with intestinal microbiota appears to be a potentially effective therapeutic strategy [11,12].

The human gut is a large, complex ecosystem that includes a diverse community of microbial species [13,14,15]. The intestinal microbiota evolves together with the host and is an integral part of the body, and colonization of a stable and diverse microbiota allows the body to maintain a healthy homeostasis [16,17]. Alterations in the intestinal microbiota not only lead to the development of intestinal diseases but also mediate the development of central nervous system diseases, with the intestinal microbiota participating in bidirectional communication with the central nervous system through metabolic, immune, neurological and endocrine pathways [18,19].

Effective interventions for intestinal microbiota include the application of single species of probiotics (e.g., *Lactobacillus* and *Bifidobacterium*) and fecal microbiota transplantation (FMT), which can improve neuroinflammatory status and reduce the deposition of Aβ plaques by recolonizing the intestinal microbiota of mice [20,21,22]. Similarly, prebiotics have shown some potential in the treatment of AD; a recent study has also shown that prebiotic lactulose can improve a cognitive deficit in AD mouse models through autophagy and anti-inflammation pathways [23]. As an alternative to probiotic supplementation, prebiotics can be used to regulate the gut microbial microbiota. Probiotics and prebiotics can be effective treatment options for neurological disorders. However, additional investigations are required to understand the underlying mechanisms in detail, considering that mere correlation does not necessarily indicate causation [22]. The application of probiotics ignores the fact that the gut is a complete microecosystem and there is competition among microorganisms [24,25,26]. Additionally, when FMT is applied to treat patients, there are some problems such as donor screening, adverse reactions, and ethical concerns [27,28]. Our laboratory isolated eight species of bacteria that occur with high frequency in the normal intestinal microbiota and defined them as intestinal dominant microbiota [29]. After preliminary study, it was found that the biological activity of these eight species of dominant bacteria was consistent with the effect of FMT and was thus expected to replace FMT in the regulation of microbiota to treat diseases. The normal colonization of intestinal microbiota has a protective function and can resist the invasion and colonization of foreign pathogenic bacteria [15]. Through previous studies, *Enterococcus* (DM9112) was confirmed by our laboratory to produce a variety of metabolites dominated by short-chain fatty acids and to have a variety of biological activities; its biological activities and safety have been fully studied. APPswe/PS1ΔE9 (APP/PS1) transgenic mice and wild type (WT) mice at eleven months of age were gavaged for five weeks with intestinal dominant microbiota, *Enterococcus* (DM9112), or normal saline. The fecal microbiota was preliminarily analyzed via polymerase chain reaction–denaturing gradient gel electrophoresis (PCR-DGGE) to identify significant changes in gut microbiota, and then the significant differences were analyzed using 16S rRNA high-throughput sequencing technology. Immunohistochemical (IHC) staining was used to observe the deposition of Aβ plaques in the hippocampus of mice. Quantitative reverse transcriptase PCR (qRT-PCR) was used to detect the relative mRNA expression levels of amyloid precursor protein (APP), β-site APP *cleaving enzyme* 1 (BACE1), interleukin-1β (IL-1β) and interleukin-6 (IL-6) in brain tissue. Western blot was used to detect the quantification of APP, BACE1, IL-1β, and IL-6 protein expression in mouse brain tissue. Serum concentrations of IL-1β and tumor necrosis factor-α (TNF-α) were determined via enzyme-linked immunosorbent assays (ELISA). It was confirmed that intestinal dominant microbiota could significantly restore the intestinal microbiota richness and composition of APP/PS1 mice to normal levels, downregulate the expression levels of IL-1β, IL-6, APP, and BACE1 in the brain, reduce their protein expression, and reduce the deposition area of Aβ plaques in the brain. Moreover, it was confirmed that the therapeutic effect of intestinal dominant microbiota was superior to that of *Enterococcus* monobacterium (DM9112). Our study provides a new therapeutic concept for AD. By adjusting the intestinal microecological balance through intestinal dominant microbiota, intestinal barrier damage can be reduced, neuroinflammation can be alleviated, and pathological deposition of Aβ plaques can be reduced; thus, intestinal dominant microbiota could replace FMT as a therapeutic approach.

## 2. Materials and Methods

### 2.1. Experimental Materials

(1) Acquisition of experimental strains

A total of 0.1 g of feces from SPF-grade C57BL/6J mice in a healthy state was collected into 1.5 mL sterile centrifuge tubes, diluted 10 times with sterile saline, and mixed thoroughly on a vortex shaker to obtain the fecal stock solution. We took 0.1 mL of the original fecal solution in 0.9 mL of sterile saline for multiplicative dilution, took 100 µL of the 10^−4^ and 10^−5^ dilutions of the bacterial solution on a preconfigured medium plate, spread it evenly with a coating stick, reversed the plate after the coating solution was completely absorbed, and carried out aerobic and anaerobic culture. *Lactobacillus reuteri*, *Enterococcus faecium*, *Escherichia coli*, *Streptococcus parasanguinis*, and *Staphylococcus nepalensis* were incubated at 37 °C for 48 h to observe the results using the feeder culture method; *Bifidobacterium animalis*, *Bacteroides ovatus,* and *Fusobacterium gastrosuis* were incubated in an anaerobic incubator at 37 °C for 72 h to observe the results (Figure S1). Primary morphological identification was then conducted by observing the colony morphology and the morphology of the bacteria under a microscope after Gram staining (Figure S2). Next, 16S-rDNA sequencing was conducted on the isolated suspicious bacteria. The sequencing results were compared with the NCBI database (Table S1), and the species information with the greatest similarity to the species sequence to be tested was defined as the strain identification result.

(2) Preparation of experimental bacterial solution

A single colony of each isolated dominant bacteria was selected and incubated in a 50 mL liquid culture medium and cultured for 24 h at 37 °C for aerobic bacteria and 48 h at 37 °C for anaerobic bacteria. Next, 0.1 mL of the bacterial growth solution of each dominant bacteria was absorbed into the new liquid culture medium of a 50 mL system, and the bacterial growth culture was continued in the same way. The dominant bacterial growth medium after the increase was counted based on the number of viable bacterial drop plates. In addition, 0.1 mL of the bacterial solution was added to 0.9 mL of sterile normal saline for multiple dilutions and finally diluted to 10^−4^, 10^−5^, and 10^−6^. Ten microliters of each dilution were dropped on a prebaked solid medium, and three parallel gradients were created for each dilution. The aerobic bacteria were cultured at 37 °C for 48 h, and the anaerobic bacteria were cultured at 37 °C for 72 h in an anaerobic incubator. Based on the countability of the colonies, a suitable dilution was then chosen for counting, and the formula for calculating the number of live bacteria was as follows: (CFU/mL) = number of colonies × dilution × 10.

Based on the table of intestinal dominant microbiota back to the instillation of bacteria (Table 1), the bacteriological enrichment solution was aspirated, centrifuged at 6000× *g* for 5 min, and the supernatant was discarded after centrifugation. After centrifugation, the pellet was washed 3 times with sterile saline and then diluted with sterile saline to a total of 0.3 mL per mouse.

### 2.2. Experimental Design

(1) Part 1: Determination of short-chain fatty acids in Enterococcus (DM9112)

A single colony of *Enterococcus* (DM9112) was selected and inoculated on a 50 mL selective liquid medium of *Enterococcus* (Table S2) and cultured at 37 °C for 24 h. Then, 1 mL of the enhanced liquid was absorbed and inoculated on a 500 mL selective liquid medium of *Enterococcus* and cultured at 37 °C for 24 h. From the beginning of the culture, 10 mL of *Enterococcus* (DM9112) fermentation solution was taken every 4 h and centrifuged at 6000× *g* and 4 °C for 8 min. After centrifugation, the supernatant was stored at −20 °C, and then the types and contents of short-chain fatty acids in the supernatant were measured via gas chromatography.

(2) Part 2: Animal experiments

APP/PS1 transgenic male mice (Better Biotechnology Co., Ltd., Guangzhou, China) coexpressing mutant human APP and PS1 on a C57BL/6J background were housed under specific pathogen-free conditions (i.e., a temperature of 24 °C, light/dark cycle of 12 h, and relative humidity of 45–65%, freely regulated). The housing of the animals and the experimental procedures were carried out in accordance with the Guide for the Care and Use of Laboratory Animals (United States National Institutes of Health) and were approved by the Dalian Medical University Animal Care Facility and Animal Research Committee (Ethics Committee approval permit No. AEE20010). This study was carried out in compliance with the ARRIVE guidelines 2.0.

As shown in Figure S3, the experimental animals were divided into four groups: *Enterococcus* (DM9112) treatment group (APP/PS1 + D), intestinal dominant microbiota treatment group (APP/PS1 + A), APP/PS1 group (APP/PS1), and wild-type control group (WT). Mice were treated intragastrically with a bacterial solution or normal saline at a dose of 0.3 mL/mouse once a day for five weeks, during which time mouse feces samples were collected and basal metabolic indices were recorded. At the end of the fifth week of treatment, the mice were sacrificed after brain or neck removal with normal saline irrigation, and fecal DNA was extracted. After PCR, the changes in fecal microbiota were preliminarily detected via DGGE. Next, 16S rRNA sequencing confirmed the changes in intestinal microbiota at the genus level (*n* = 6). Frozen sections of brain tissue were prepared for IHC staining to observe the deposition of Aβ plaques in the brain (*n* = 3). The relative expression levels of APP, BACE1, IL-1β, and IL-6 mRNA in brain tissues were measured via qRT-PCR (*n* = 4). Meanwhile, the Western blot technique was applied to detect APP, BACE1, IL-1β, and IL-6 protein expression in brain tissues (*n* = 3). Serum levels of IL-1β and TNF-α were determined via ELISA (*n* = 3–4) (Table S3). 

### 2.3. DNA Extraction and Polymerase Chain Reaction-Denaturing Gradient Gel Electrophoresis (PCR-DGGE)

Mouse fecal samples were collected, and all samples were stored at −80 °C for backup, followed by the application of (E.Z.N.A.^®^ Stool DNAkit, Omega kit, Norcross, GA, USA) for the extraction of genomic DNA. For the extracted DNA samples, the V3 region of 16S rRNA was amplified using primers 338F-806R (338F, 5′-CGCCCGGGGCGCGCCCCGGGCGGGGCGGGCACGGGGGGCCTACGGGAGGCAGCAG-3′ and 518R, 5′-ATTACCGCGGCTGCTGG-3′). The PCR system contained 25 µL PCR Master Mix, 1 µL forward primer (10 Pmol/µL), 1 µL reverse primer (10 Pmol/µL), and 3 µL DNA, and the total system was adjusted to 50 µL using enzyme-free water. The concentration of DNA was determined using a NanoDrop2000 (Thermo Scientific, Waltham, MA, USA) and DNA was stored at −20 °C. The PCR-amplified products were transferred to a DGGE gel, placed in a 1× TAE solution, and electrophoresed at a constant temperature and pressure of 60 °C and 80 V, respectively, for 6 h. After electrophoresis, the gel was stained with EB for 30 min, and the pictures were developed with a UVI fully automated gel imaging system (Bio-Rad, Hercules, CA, USA). DNA fragments of different sequences were separated via PCR-DGGE; the bands at different positions represent different microorganisms.

### 2.4. Microbiota Analysis by 16S rRNA Sequencing

The 16S amplicons were purified with a GeneJET PCR Purification Kit (Thermo Fisher Scientific, Waltham, MA, USA). A DNA library was constructed using the Ion Plus Fragment Library Kit 48 rxns (Thermo Fisher Scientific, Waltham, MA, USA). After the library was quantified with a Qubit fluorometer (Qubit 3.0, Invitrogen, Carlsbad, CA, USA) and qualified, it was sequenced by an Ion S5 XL system (Thermo Fisher Scientific, Waltham, MA, USA).

### 2.5. Bioinformatic and Statistical Analysis

Quality filtering was performed on the raw reads to obtain high-quality clean reads. According to Cutadapt (v1.9.1) (http://cutadapt.readthedocs.io/en/stable/, accessed on 10 July 2023), the reads were compared with the GOLD reference database (http://drive5.com/uchime/uchime_download.html, accessed on 10 July 2023) with the UCHIME algorithm (http://www.drive5.com/usearch/manual/uchime_algo.html, accessed on 10 July 2023) to detect and remove chimaeric sequences to obtain clean reads.

Sequence analysis was performed with UPARSE software (Uparse v7.0.1001) (http://drive5.com/uparse/, accessed on 10 July 2023). Sequences with ≥97% similarity were assigned to the same operational taxonomic units (OTUs). Representative sequences for each OTU were screened for further annotation. For each representative sequence, the SSU rRNA database of Silva (http://www.arb-silva.de/, accessed on 10 July 2023) was used based on the Mothur algorithm to annotate taxonomic information (set threshold from 0.8 to 1). For determination of the phylogenetic relationships of different OTUs and the difference in the dominant species in different samples (groups), multiple sequence alignments were conducted using MUSCLE (http://www.drive5.com/muscle/, accessed on 10 July 2023) software (v3.8.31). OTUs abundance information was normalized using a standard sequence number corresponding to the sample with the fewest sequences. Subsequent analyses of alpha diversity and beta diversity were all performed based on these output normalized data.

The Alpha diversity indexes Observed_species, Shannon and PD_whole_tree were calculated using Qiime software (Version 1.9.1) and analyzed using R software (V2.15.3, http://www.R-project.org, accessed on 10 July 2023). Beta diversity was calculated using the weighted UniFrac distance. Principal component analysis (PCA) based on the UniFrac distance was performed using R software. NMDS analysis was based on Bray–Curtis dissimilarity and performed using the vegan software package of R software. Tukey’s test was applied to perform post hoc tests, with *p* < 0.05 considered a significant difference. R software was used for permutational multivariate analysis of variance (Adonis) to analyze the between-group differences in beta diversity.

### 2.6. Immunohistochemical Staining

Mouse brains were fixed with 4% paraformaldehyde and embedded after dehydration with 20% and 30% sucrose solutions. A cryostat (Leica CM 1850, Leica Microsystems AG, Wetzlar, Germany) was used to obtain continuous 10 μm sections of the brains in the coronal plane. The sections were subsequently incubated with rabbit anti-Aβ (1:200; 6E10, BioLegend, San Diego, CA, USA) supplemented with 5% normal goat serum and 0.05% Triton X-100 overnight at 4 °C. The number and area ratio of positive depositions of Aβ plaques in the hippocampal region was determined from three slices of each brain sectioned in the cephalic body and caudal area, and a microscopic imaging system (Nikon, Tokyo, Japan) was used to capture the images. The images were analyzed using Image-Pro Plus 6.0 software (Media Cybernetics, Rockville, MD, USA).

### 2.7. Quantitative Reverse Transcriptase Polymerase Chain Reaction

All RNA from brain tissue was extracted using TRIzol reagent (TransGen Biotech, Beijing, China), cDNA was synthesized using a RevertAid First Strand cDNA Synthesis Kit (Thermo Scientific, Waltham, MA, USA), and 20 μL of solution was used for amplification. The system included 10 μL TB Green Premix Ex Taq™ II (Tli RNaseH Plus), 0.4 μL PCR forward primer, 0.4 μL PCR reverse primer, 2 μL cDNA, and 7.2 μL ddH_2_O. The PCR thermal cycling parameters were: 95 °C for 30 s, 95 °C for 15 s, and 60 °C for 30 s to collect the fluorescence signal (for 40 cycles), and the results were used to calculate the relative expression of mRNA using the 2^−ΔΔCT^ method. The primers involved are detailed in Table S4.

### 2.8. Western Blot Analysis

To prepare whole extracts, frozen tissues (from the cortex and hippocampus) were homogenized in a lysis buffer and protease inhibitor (Roche Diagnostics, Indianapolis, IN, USA). After ultracentrifugation (150,000× *g*, 4 °C, 45 min), the supernatants were collected and stored at −80 °C for future use. All samples were subjected to a Bradford protein assay. Equal amounts of protein were separated on acrylamide gels and transferred onto polyvinyl difluoride (PVDF) membranes. Western blotting was performed under standard conditions, applying rabbit polyclonal antibodies against APP (1:1000; Proteintech, San Diego, CA, USA), BACE1 (1:800; Proteintech), IL-1β (1:1000; Proteintech), IL-6 (1:1000; Proteintech), β-tubulin (1:1000; Protein biology) at 4 °C overnight. The latter antibody was used for loading normalization. The anti-rabbit peroxidase-conjugated secondary antibodies were applied at 1:5000 for 1 h at room temperature and blots were visualized with an ECL detection kit (Amersham, Buckinghamshire, UK). Images obtained were quantified using the ImageJ v 1.8.0 software.

### 2.9. Measurement of Serum Pro-Inflammatory Factor (IL-1β and TNF-α) Concentrations

Based on the protocols of the ELISA kit (Jiangsu Enzyme Label Biotechnology Co., Ltd., Nanjing, China), 50 μL of standards of different concentrations, 40 μL of sample dilution, and 10 μL of serum were added to the sample wells. Following enzyme addition, warming, liquid preparation, washing, color development, and termination of color development, the absorbance (OD) of each well at 450 nm was measured using an enzyme marker (Thermo Scientific, Waltham, MA, USA).

### 2.10. Statistical Analysis

GraphPad Prism 8 software and SPSS 19.0 software were used for statistical analysis, and all values are expressed as the mean ± SEM. Data between multiple groups were analyzed using a one-way analysis of variance (ANOVA), followed by an LSD post hoc test or Dunnett post hoc test, depending on whether the variance was chi-square. A *p* value < 0.05 was considered statistically significant (* *p* < 0.05, ** *p* < 0.01, *** *p* < 0.001).

## 3. Results

### 3.1. Changes of Fatty Acid Types and Contents of Metabolites of Enterococcus (DM9112)

**Figure 1:**
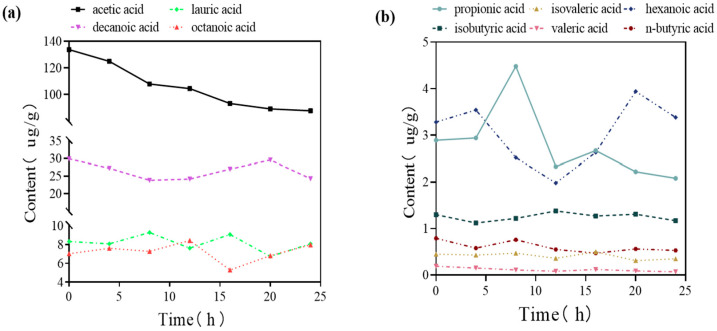
In vitro experiments were performed to detect the trend of the content of each short-chain fatty acid in the metabolites of *Enterococcus* (DM9112) over time. (**a**) Acetic acid, decanoic acid, octanoic acid and lauric acid contents. (**b**) Propionic acid, isobutyric acid, valeric acid, isovaleric acid, hexanoic acid, and n-butyric acid contents.

Short-chain fatty acids produced by intestinal microbiota have been indicated to have certain anti-inflammatory effects and act on the central nervous system through metabolic pathways, thus playing a role in nerve protection [30]. We determined via in vitro experiments that *Enterococcus* (DM9112) metabolites were present, and we detected ten short-chain fatty acids: acetic acid, propionic acid, isobutyric acid, n-butyric acid, isovaleric acid, hexanoic acid, decanoic acid, octanoic acid, valeric acid and lauric acid. The content of acetic acid was the highest among the SCFAs, and its content gradually decreased over a 24 h period. Our analysis showed that acetic acid gradually decreased as the fermentation time increased. The reason for this may be that *Enterococcus* (DM9112) used glucose for energy supply in the initial stage of fermentation, but glucose was consumed with the increase in time, so acetic acid as a carbon source continued to be used as fuel for the energy supply. The contents of decanoic acid, lauric acid, and octanoic acid were the next highest, and these contents did not change significantly in 24 h (Figure 1a). The contents of propionic acid, isobutyric acid, n-butyric acid, isovaleric acid, valeric acid, and hexanoic acid were very low; among these, propionic acid showed a significant increase at 8 h and then decreased and subsequently stabilized, whereas the contents of the other fatty acids showed no significant trend over a 24 h period (Figure 1b).

### 3.2. The Intestinal Microbiota of APP/PS1 Mice Showed an Increasing Trend in the Bacteroides Phylum and a Decreasing Trend in the Firmicutes Phylum

**Figure 2:**
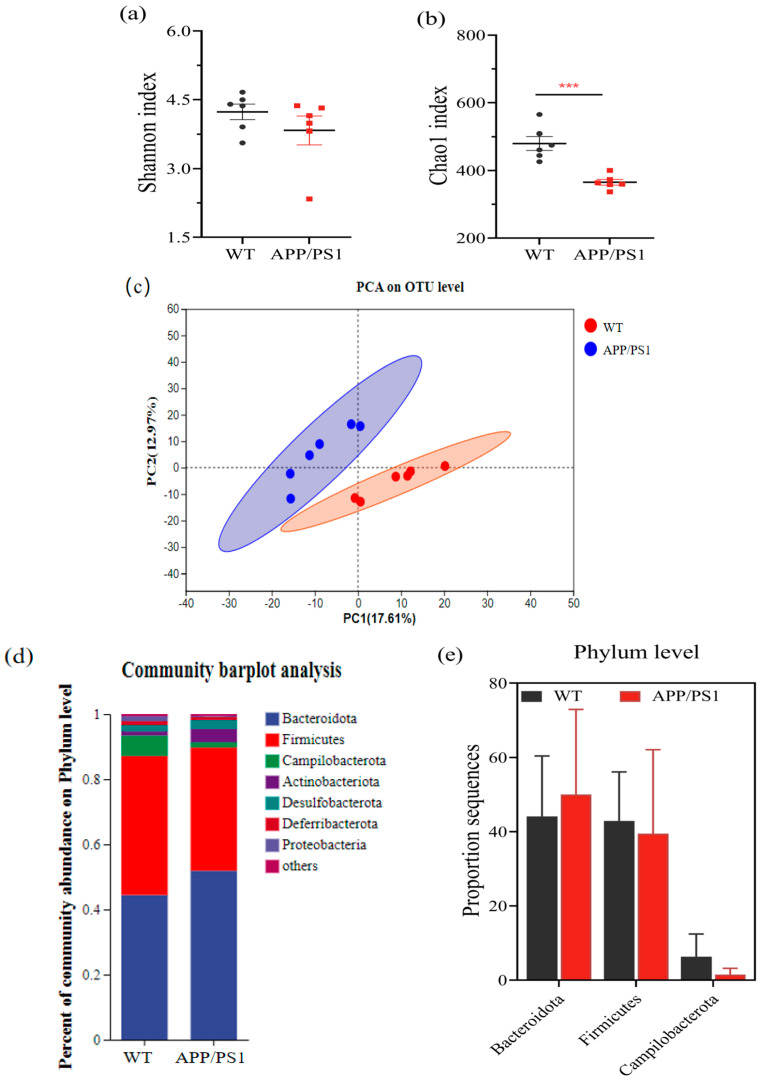

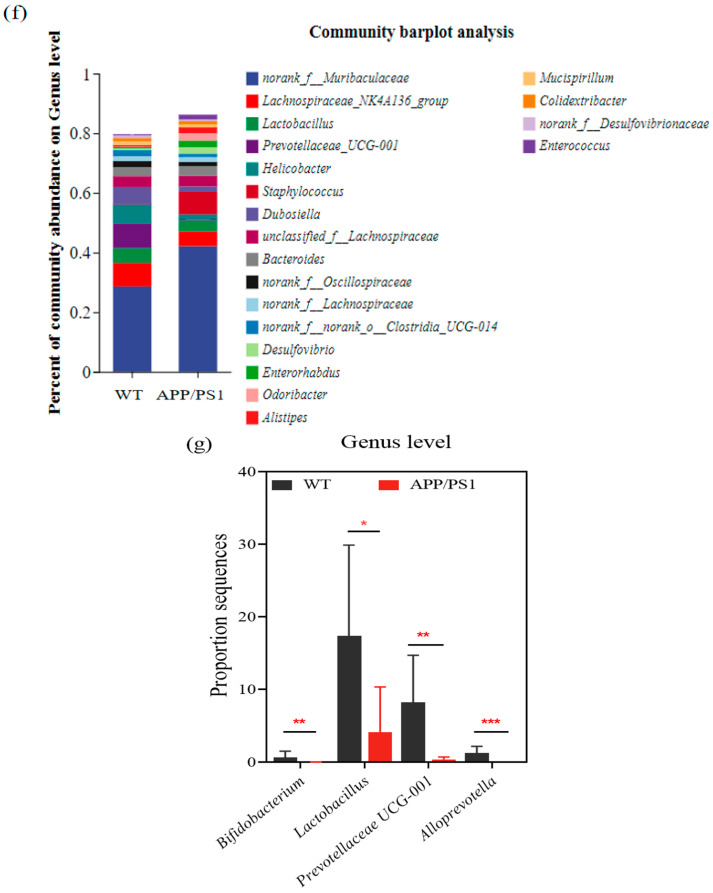
Comparison of intestinal microbiota composition between APP/PS1 mice and WT mice at eleven months of age. (**a**) Microbial community diversity. (**b**) Microbial community richness. (**c**) Principal component analysis diagram. (**d**) Histogram of horizontal community composition at the phylum level. (**e**) Histogram of phylum-level dominant microbiota differences. (**f**) Histogram of horizontal community composition at the genus level. (**g**) Bar chart of the difference in dominant microbiota at the genus level. Values represent the mean ± S.E.M. * *p* < 0.05, ** *p* < 0.01. *** *p* < 0.001. *n* = 6 mice/group.

To determine the specific changes in intestinal microbiota, we sequenced the 16S rRNA gene in the feces of 11-month-old APP/PS1 and WT mice. APP/PS1 mice had significantly lower intestinal microbiota richness and diversity than WT mice (Figure 2a,b). Differences in the composition of the gut microbiota were also observed between the two groups based on an unweighted Unifrac principal component analysis (PCA) (Figure 2c). At the phylum level, Bacteroidetes, Firmicutes, and Campilobacterota were the dominant taxa. Compared with those of WT mice, the richness of Bacteroidetes in APP/PS1 mice increased, whereas the abundances of Firmicutes and Campilobacterota decreased (Figure 2d,e). At the genus level, *Bacteroides*, *Lachnospiraceae_NK4A136_group*, *Lactobacillus,* and *Prevotellaceae_UCG_001* dominated. Compared with those of WT mice, the abundances of *Bifidobacterium*, *Lactobacillus*, *Prevotellaceae UCG-001*, *Allprevotella,* and *Helicobacter* decreased significantly in APP/PS1 mice (Figure 2f,g).

### 3.3. Intestinal Dominant Microbiota in APP/PS1 Mice at the End of the Second Week of Treatment Restored the Intestinal Microbiota Composition and Richness to That of Control Mice at the Same Age

**Figure 3:**
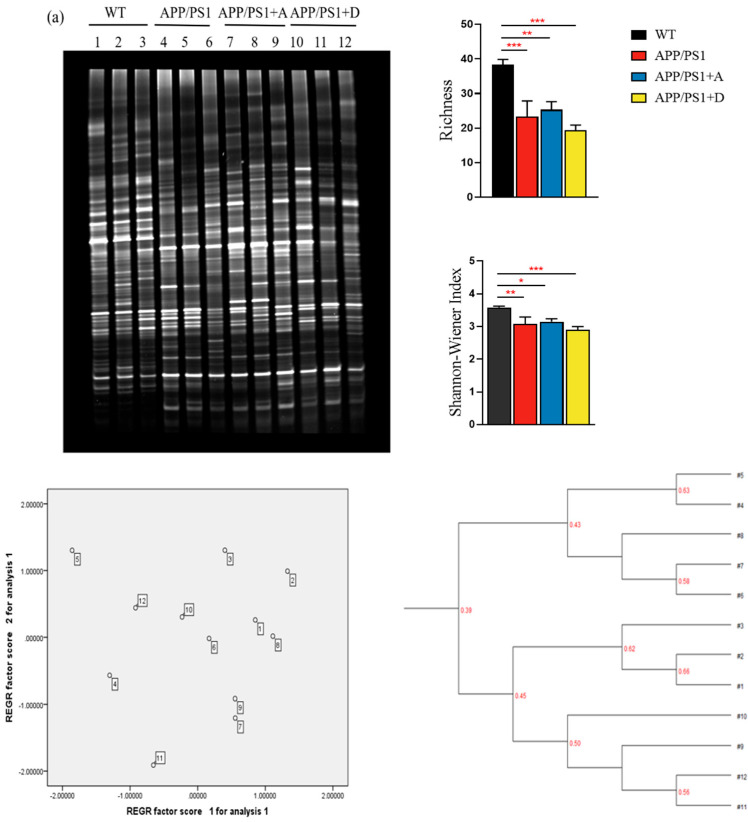

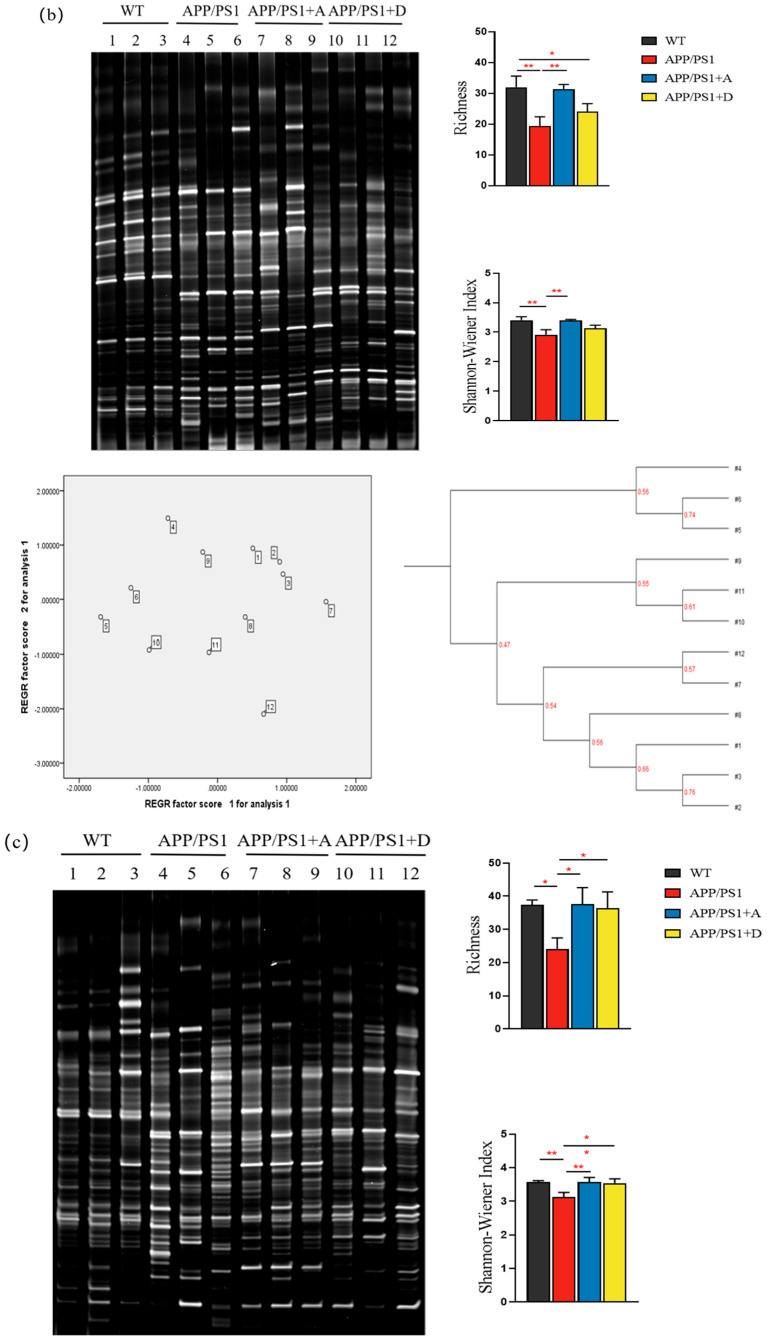

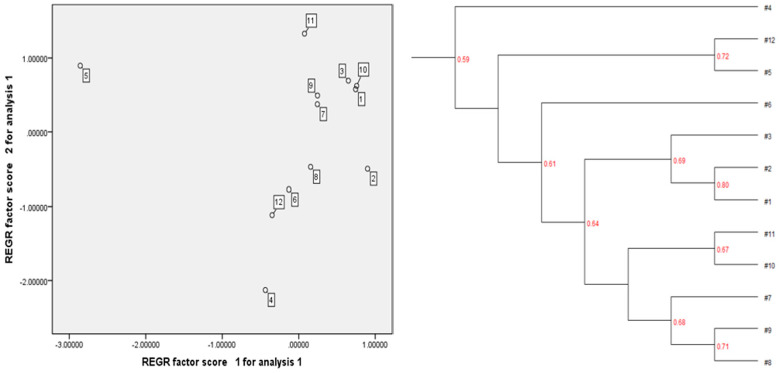
PCR-DGGE imaging at different intervention periods. Electrophoresis gels (different bands represent different microorganisms), bar charts of richness and the Shannon index, cluster analysis diagrams and principal component analysis diagrams comparing the different treatments. (**a**) The initial stage before treatment. (**b**) The second week of treatment. (**c**) The fifth week of treatment. Values represent the mean ± S.E.M. * *p* < 0.05, ** *p* < 0.01, *** *p* < 0.001. *n* = 3 mice/group.

Polymerase chain reaction-denaturing gradient gel electrophoresis (PCR-DGGE) is a proven technique for the detection of intestinal or fecal microbiota. It is characterized by high sensitivity and speed [31]. Therefore, PCR-DGGE was used to preliminarily observe the change nodes of the mouse intestinal microbiota. At the early stage of intervention, the intestinal microbiota richness and Shannon index of APP/PS1 mice were significantly decreased compared with those of WT mice of the same age. The cluster analysis diagram clearly shows two clusters, and the principal component analysis diagram also shows distinct regions. There were also significant differences in the composition of the microbiota (Figure 3a). At the end of the first week of treatment, the abundance of intestinal microbiota and Shannon index of mice in both treatment groups showed a certain trend of recovery compared to APP/PS1 mice (Figure S4a). At the end of the second week of treatment, the intestinal microbiota richness and Shannon index of the gut-dominant microbiota treatment group (APP/PS1 + A) mice were basically restored to the levels of WT mice of the same age (Figure 3b). At the end of the third week of treatment, the intestinal microbiota richness and Shannon index of the *Enterococcus* (DM9112)-treated group (APP/PS1 + D) mice had basically recovered to the levels of WT mice of the same age. These results indicate that the administration of intestinal dominant microbiota could restore the intestinal microbiota level of APP/PS1 mice (Figure S4b). In the results of the cluster analysis in the fourth week, the APP/PS1 + A and APP/PS1 + D mice were completely clustered with the WT mice and were completely separated from APP/PS1 mice (Figure S4c). By the fifth week of treatment, the richness and composition of the intestinal microbiota of APP/PS1 + A and APP/PS1 + D mice were both significantly recovered (Figure 3c).

### 3.4. Enterococcus (DM9112) and Intestinal Dominant Microbiota Were Able to Restore Intestinal Microbiota Diversity and Increase Colonization of Beneficial Bacteria in APP/PS1 Mice by the End of the Fifth Week

**Figure 4:**
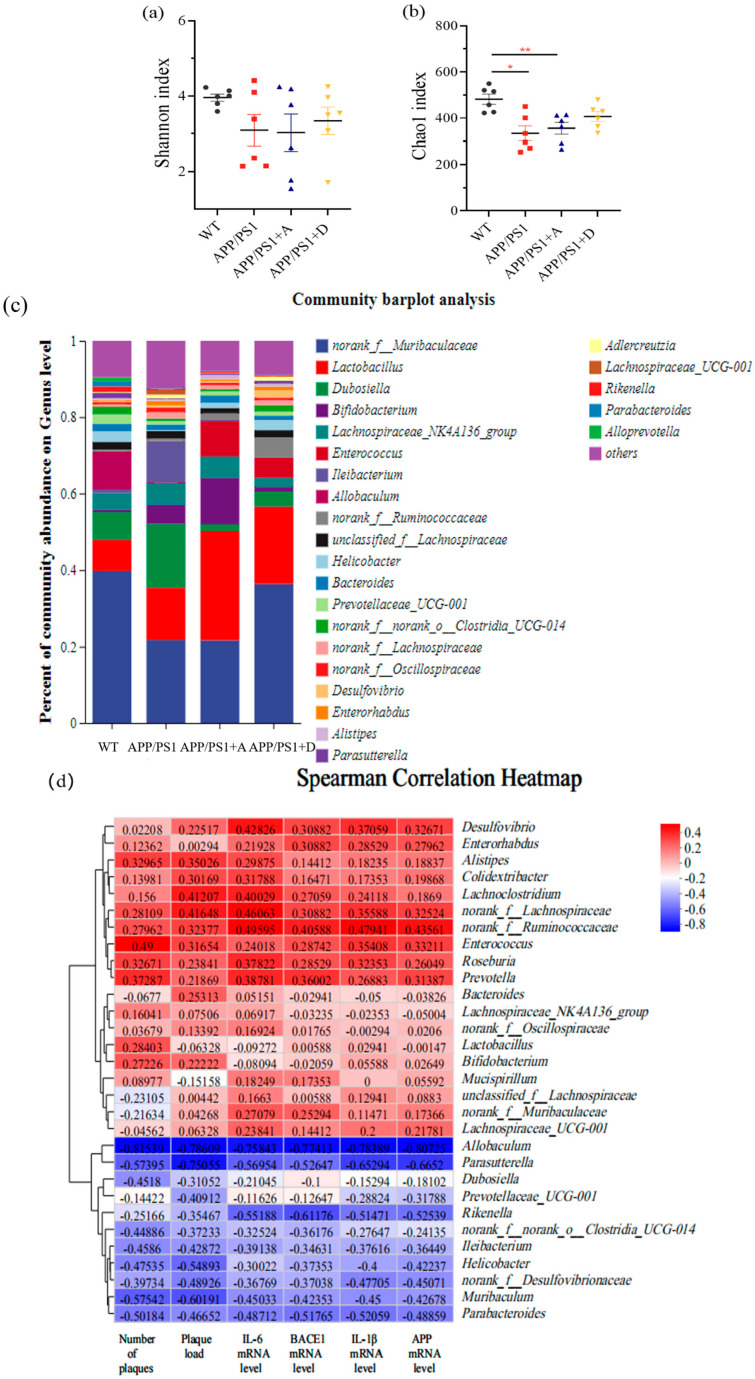
Changes in the intestinal microbiota of mice treated with *Enterococcus* (DM9112) and dominant intestinal microbiota via gavage for five weeks. (**a**) Microbial community diversity. (**b**) Microbial community richness. (**c**) Histogram of horizontal community composition at the genus level. (**d**) Clinical factor association analysis. Values represent the mean ± S.E.M. * *p* < 0.05, ** *p* < 0.01. *n* = 6 mice/group.

Once again, we applied 16S rRNA gene sequencing technology to explore the changes in intestinal microbiota in mice after five weeks of treatment with *Enterococcus* (DM9112) and intestinal dominant microbiota. Compared with mice in the WT group, the intestinal microbiota diversity of mice in APP/PS1 + A and APP/PS1 + D groups was significantly restored (Figure 4a). The intestinal microbiota richness of APP/PS1 + D mice also recovered significantly, but the intestinal microbiota richness of APP/PS1 + A mice still did not return to normal levels (Figure 4b). However, in non-metric multidimensional scaling (NMDS) analysis, APP/PS1 + A, APP/PS1 + D, and WT mice did not show significant clustering of microbiota (Figure S5a). The abundance of *Lactobacillus*, *Bifidobacterium*, *Helicobacter*, *Bacteroides,* and *Enterococcus* increased and the abundance of *norank_f_Oscillospiraceae* and *Lachnospiraceae_UCG-001* decreased compared with APP/PS1 + A, as determined by differential abundance analysis at the genus level of bacterial taxa; there was increased abundance of *Lactobacillus*, *Helicobacter,* and *Enterococcus* and decreased abundance of *Bifidobacterium*, *norank_f_Oscillospiraceae,* and *Lachnospiraceae_UCG-001* compared with APP/PS1 + D (Figure 4c and Figure S5b). A clinical factor association analysis was also performed (Figure 4d) to demonstrate the potential association between bacterial abundance and mice phenomes. We found that the enrichment of *Bifidobacterium* was negatively correlated with the expression levels of IL-6 and BACE1 mRNAs in brain tissue, and the abundance of *Lactobacillus* was negatively correlated with the deposition area of Aβ plaques in the brain hippocampus. The enrichment of *Bacteroides* was negatively correlated with the number of deposited Aβ plaques in the brain hippocampus, and the abundance of *Helicobacter* was negatively correlated with the expression levels of IL-6, IL-1β, BACE1 and APP mRNAs in brain tissue. The abundance of *Lachnospiraceae_UCG-001* was positively correlated with brain tissue IL-6, IL-1β, APP, and BACE1 mRNA expression levels, and the enrichment of *norank_f_Oscillospiraceae* was positively correlated with the number and area of brain hippocampal Aβ plaques deposited and brain tissue IL-6, APP and BACE1 mRNA expression levels.

### 3.5. The Reinfusion of Enterococcus (DM9112) and the Dominant Intestinal Microbiota Significantly Restored the Abundance of Lactobacillus and Bifidobacterium to Normal Levels

**Figure 5:**
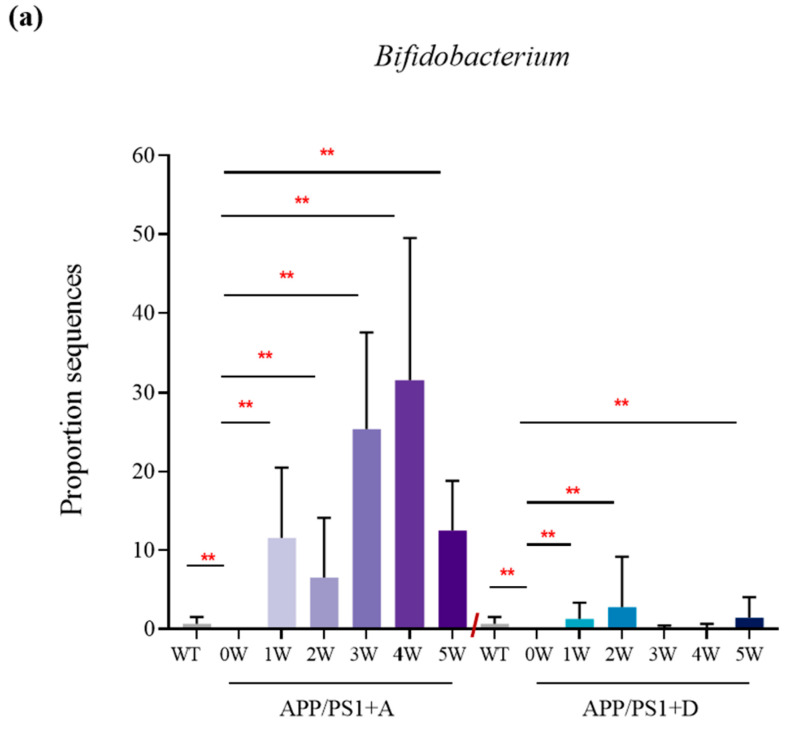

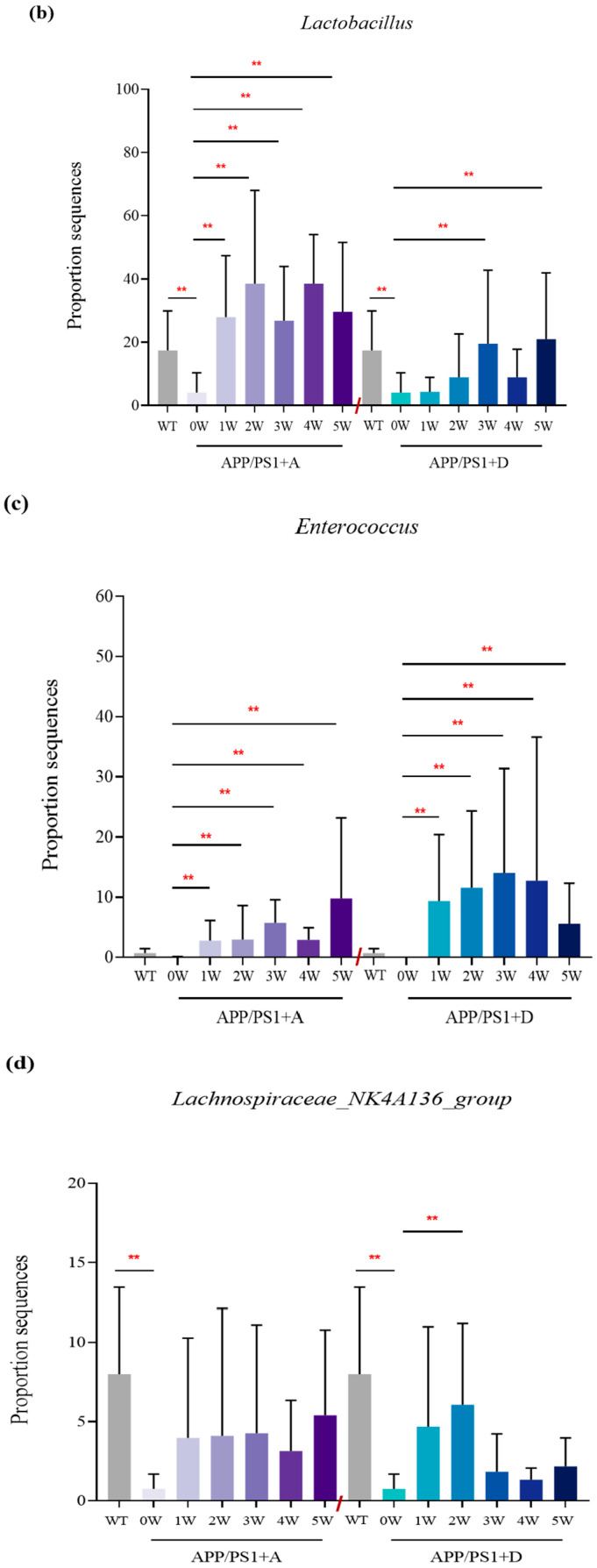

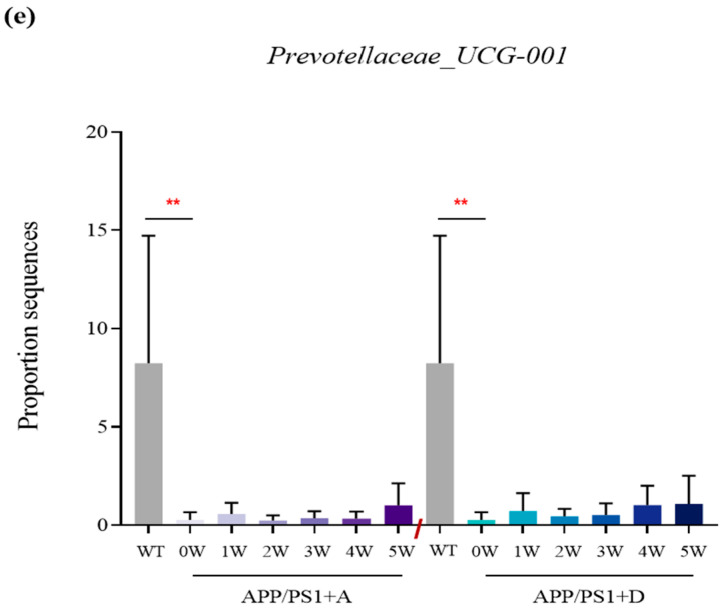
Colony enrichment of APP/PS1 + A and APP/PS1 + D mice compared with that of WT (0 W) and APP/PS1 (0 W) mice at different time points (0 W, 1 W, 2 W, 3 W, 4 W, and 5 W). (**a**) *Bifidobacterium,* (**b**) *Lactobacillus,* (**c**) *Enterococcus,* (**d**) *Lachnospiraceae_NK4A136_group,* and (**e**) *Prevotellaceae_UCG_001* Values represent the mean ± S.E.M. ** *p* < 0.01. *n* = 6 mice/group.

The abundances of *Bifidobacterium*, *Lactobacillus*, *Lachnospiraceae_NK4A136_group*, and *Prevotellaceae_UCG_001* were significantly reduced by APP/PS1 compared with those of WT, and the abundances of *Bifidobacterium* and *Lactobacillus* in APP/PS1 + A were significantly restored at the end of the fifth week (Figure 5a,b) and significantly increased and exceeded the normal level for *Enterococcus* with the increase in the treatment cycle (Figure 5c). In APP/PS1 + D, the abundance of *Lachnospiraceae_NK4A136_group* was significantly increased in the second week of treatment, the *Lactobacillus* abundance recovered significantly in the third week of treatment (Figure 5d), and the *Bifidobacterium* abundance was low during the fourth week of treatment. However, at the end of the fifth week, the abundance of *Lactobacillus* and *Bifidobacterium* recovered significantly, and the abundance of *Enterococcus* increased significantly and exceeded the normal level. However, the abundance of *Prevotellaceae_UCG_001* was not restored to normal levels in either treatment group (Figure 5e).

### 3.6. Reinfusion of Enterococcus (DM9112) and Intestinal Dominant Microbiota Reduced the Deposition Area of Aβ Plaques and Reduced Brain Inflammation in the Hippocampus of APP/PS1 Mice

**Figure 6:**
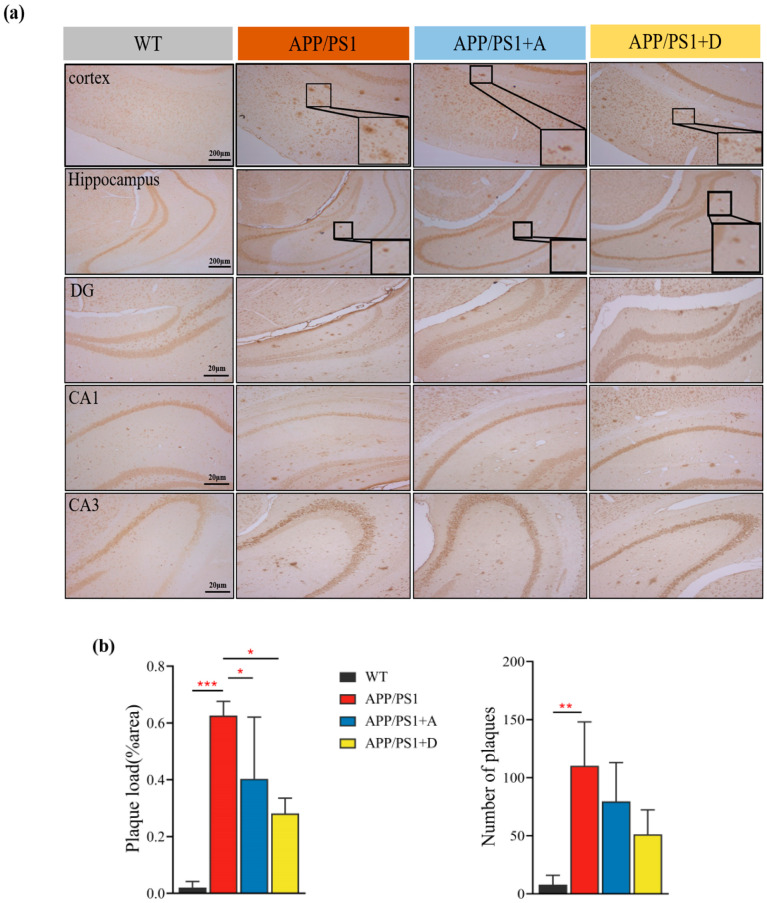

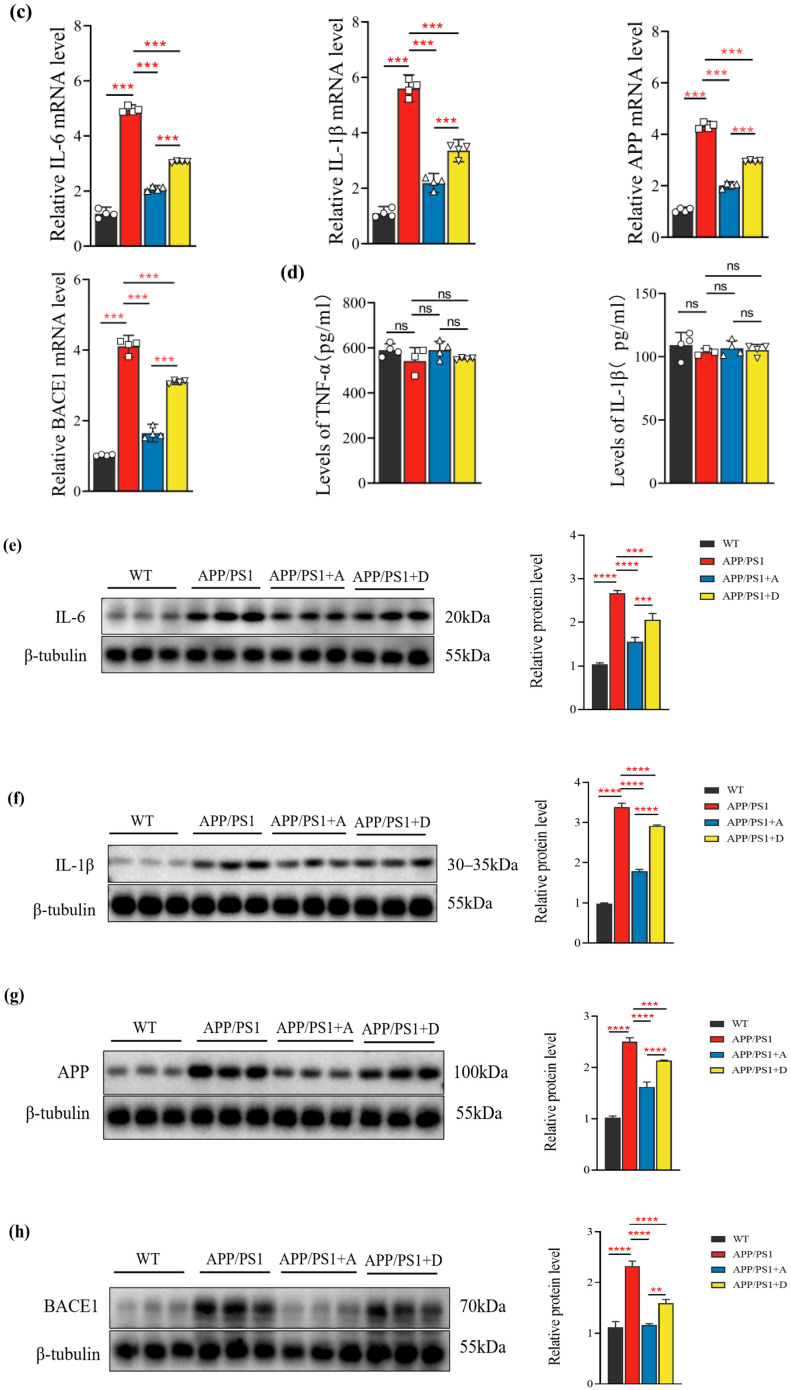
Brain amyloid pathology, neuroinflammation and serum inflammation in four groups of mice. (**a**) IHC staining of brain hippocampal coronal sections with anti-Aβ antibody; scale bars are 10 μm and 50 μm (*n* = 3 mice/group). (**b**) Quantification of the size and number of Aβ plaque areas in the hippocampus (*n* = 3 mice/group, 9 slices per mouse were selected for counting). (**c**) Expression levels of IL-6, IL-1β, APP and BACE1 mRNA in brain tissue (*n* = 4 mice/group). (**d**) Serum concentrations of TNF-α and IL-1β were determined via ELISA (*n* = 3–4 mice/group). (**e**–**h**) Western blot for detection of IL-6, IL-1β, APP and BACE1 protein expression levels in brain tissue and quantitative analysis (*n* = 3 mice/group). Values represent the mean ± S.E.M. * *p* < 0.05, ** *p* < 0.01, *** *p* < 0.001, **** *p* < 0.0001, ns *p* > 0.05.

The main pathological manifestations of AD are the deposition of amyloid plaques and tangles of nerve fibers [5,32]. Recent studies have shown that pathological accumulation of Aβ is a key factor driving the inflammatory response of AD nerves [33], but the mechanism of action remains unclear. To investigate whether changes in the intestinal microbiota of APP/PS1 mice affect the pathological deposition of Aβ plaques, we first performed IHC staining of coronal sections with anti-Aβ antibodies and selected nine sections per mouse for statistical comparison of the proportional area and number of Aβ plaques. We found that the reinfusion of intestinal dominant microbiota and *Enterococcus* (DM9112) significantly reduced the proportional area of Aβ plaques in the hippocampus, and the number of Aβ plaques showed a decreasing trend (Figure 6a,b). qRT-PCR analysis also confirmed these results, and the APP/PS1 + A and APP/PS1 + D groups had significantly downregulated APP and BACE1 mRNA expression levels (Figure 6c). Western blot was used to confirm that APP and BACE1 protein expression levels were reduced in brain tissue (Figure 6g,h). Compared with that of *Enterococcus* (DM9112), the effect of intestinal dominant microbiota was more significant. Therefore, we concluded that intestinal dominant microbiota and *Enterococcus* (DM9112) reduce the accumulation of Aβ plaques by inhibiting BACE1 cleavage of APP. With regard to neuroinflammation, we confirmed that the mRNA expression levels of proinflammatory factors (IL-6 and IL-1β) in the brain tissue of the APP/PS1 + A and APP/PS1 + D groups were decreased (Figure 6c) and its protein expression level was also significantly reduced (Figure 6e,f). More importantly, the therapeutic effect of APP/PS1 + A was more significant. Serum proinflammatory factors (IL-6 and IL-1β) were measured via ELISA, and the effect of the two treatment groups was not significant compared with that of APP/PS1 (Figure 6d).

## 4. Discussion

APP/PS1 mice were treated with intestinal dominant microbiota or *Enterococcus* (DM9112) for five weeks, and the results showed that both treatments restored the richness and composition of intestinal microbiota, increased the colonization of beneficial bacteria, and reduced neuroinflammation. Aβ deposition was reduced by downregulating BACE1, a key rate-limiting enzyme of Aβ production, and the Aβ precursor APP. The recovery effect of intestinal dominant microbiota was faster and superior to that of *Enterococcus* (DM9112). *Enterococcus* (DM9112) is a bioactive strain isolated in our laboratory, and its metabolites contain a high yield of short-chain fatty acids. Compared with strains of high and equal quantity such as *Bifidobacterium*, *Enterococcus* was intermediate in abundance in the intestinal tract in multiple studies (the content of *Bifidobacterium* in feces was approximately 10^10^–10^11^ CFU/g, and the content of *Enterococcus* was approximately 10^7^–10^8^ CFU/g). Thus, *Enterococcus* was more capable of stabilizing the intestinal microecosystem and may be the main stable strain restricting other intestinal microbiota [34,35]. Our study provides a new perspective on the treatment of AD. The therapeutic effect of intestinal dominant microbiota is superior to that of single probiotics, and the treatment of intestinal dominant microbiota could perhaps become an alternative therapy for FMT.

The etiology of AD is not clear, but its main pathology is the deposition of amyloid plaques and tangles of nerve fibers, in addition to the proliferation of dystrophic neuropil, neuronal lines, and astrocytes, as well as the activation of microglia [5]. AD is also accompanied by an inflammatory state of the central nervous system. The human gut contains a large number of microbial species that form a complex consortium within the organism [13], and the gut microbiota evolves in concert with the growth and development of the host; it is an integral part of the human body and is considered an “organ” of the body that contributes to maintaining healthy homeostasis [16]. Recent studies have shown that alterations in intestinal microbiota can lead to the development of not only enterogenic diseases such as infections, but also central nervous system disorders [18,36,37], the mechanisms of which are currently thought to exhibit bidirectional communication with the central nervous system through metabolic, immune, neural, and endocrine pathways [19,38].

Effective interventions for gut microbiota include the application of probiotics and other measures such as fecal microbiota transplantation (FMT) [39,40]. Goyal and Ali found that the progression of clinical symptoms of AD was effectively delayed by oral probiotic treatment of AD when the intestinal microbiota disorder was corrected and the probiotics were able to induce neurotransmitter changes via neuronal pathways [40]. Bravo et al. demonstrated that long-term application of *Lactobacillus rhamnosus* reduced GABA mRNA expression in the prefrontal cortex and amygdala, decreased stress-induced corticosterone levels, and affected anxiety- and depression-related behaviors [21]. The aforementioned studies suggest that the intestinal microbiota has an ameliorative effect on the pathology and behavior of AD. However, thus far, researchers have focused on single strains of intestinal microbiota such as *Bifidobacterium* and *Lactobacillus*, ignoring the fact that the intestinal microbiota exists as a complete microecological system in the intestine. There are more than a thousand species of microbes in the intestinal microecological system. Interactions among microbes, between microbes and hosts, and between hosts and the external environment all have an impact on the organism, and the combined interactions between microbes are particularly important [41]. FMT is the transplantation of functional microbiota from healthy human feces into the gastrointestinal tract of patients for the purpose of reconstituting the intestinal microbiota [27]. Sun et al. evaluated the therapeutic effect of FMT on AD and found that FMT was effective in improving cognitive deficits and reducing brain deposition of Aβ in APP/PS1 mice, and these improvements were accompanied by reductions in tau protein phosphorylation and Aβ40 and Aβ42 levels and reversed changes in intestinal microbiota and SCFAs [39]. However, FMT does not appear to be fully applicable to clinical treatment as it is still flawed due to issues of donor screening, adverse effects, safety, and ethics [27]. This makes it particularly urgent to find better therapeutic measures compared with FMT. Our previous study found that there is a dominant intestinal microbiota, that the dominant microbiota is superior when it comes to stabilizing the intestinal microecosystem, and that there is a paucity of studies that apply a combination of intestinal microbiota to affect AD. Therefore, this experiment selected eight dominant intestinal bacteria in mice to study their relationship with Aβ plaques in the brain hippocampus and neuroinflammatory factors in AD. We applied the APP/PS1 mouse model to explore the impact of intestinal dominant microbiota in improving Aβ plaques in the brain hippocampus and inflammatory factors in the brain and circulating blood to provide new ideas and lay the foundation for the clinical treatment of AD.

It was found that the abundance and diversity of fecal microbiota of APP/PS1 mice were significantly lower than those of WT mice at the same age, and APP/PS1 and WT mice were partitioned in significantly different parts of the principal component analysis graph, which demonstrates significant differences in the composition of intestinal microbiota. Bacteroidetes abundance decreased and Firmicutes abundance increased in the intestinal microbiota of APP/PS1 mice, which is consistent with the findings of Vogt et al. [9]. The abundances of some beneficial bacteria such as *Bifidobacterium*, *Lactobacillus*, *Lachnospiraceae_NK4A136_group*, *Prevotellaceae UCG-001*, *Alloprevotella,* and *Helicobacter* were significantly reduced. *Bifidobacterium* can produce the brain neurotransmitter gamma amino butyric acid (GABA) [42], which is also associated with tryptophan metabolism and improves AD through an immune–microbial interaction [43]. Both *Bifidobacterium* and *Lactobacillus* produce short-chain fatty acids, which are important molecules that are used by the body to mediate energy signals, inhibit the growth of other harmful bacteria in the intestine, and are associated with microglial cell maturation [44,45]. At the end of the second week of treatment with *Enterococcus* (DM9112), the abundance of *Lachnospiraceae_NK4A136_group*, an important digestive bacterium in humans that can hydrolyze starch and many sugars and produce butyrate and short-chain fatty acids, was restored in the fecal microbiota of APP/PS1 mice [10]. Additionally, the abundance and diversity of microbiota flora were significantly restored. At the end of the fifth week of treatment, the diversity and abundance of fecal microbiota were significantly restored in both treatment groups, and the abundances of both *Bifidobacterium* and *Lactobacillus* were restored to the same levels as those in WT mice of the same age before treatment. The reinfusion of the dominant intestinal microbiota increased the colonization of beneficial bacteria *Helicobacter* and *Bacteroides*, and we observed that *Helicobacter* and *Bacteroides* were negatively correlated with the relative expression levels of neuroinflammatory cytokines (IL-1β and IL-6), APP and BACE1 mRNA, which is consistent with the results of Zhuang et al. [10], and reduced the enrichment of *norank_f_Oscillospiraceae* and *Lachnospiraceae_UCG-001*, which were shown to promote the deposition of Aβ plaques in the brain hippocampus and exacerbate neuroinflammation. Lower fecal microbiota richness and diversity were associated with reinfusion of the dominant intestinal microbiota, possibly due to an alteration in the composition of the intestinal microbiota and/or a reduction in the intestinal microbiota as a result of competition between microbes [46]. Why a polybacteria introduction would change the intestinal microecosystem and lead to a decrease in intestinal microbial diversity remains to be further studied in relation to the intestinal microecosystem structure of AD mice.

The impact of AD is not limited to neuronal regions but also involves the role of immune mechanisms in the central nervous system, where misfolded and aggregated proteins bind to pattern recognition receptors on microglia and astrocytes and trigger innate immune responses characterized by the release of inflammatory mediators, which contribute to disease progression and severity. The composition of gut microbes can also alter the permeability of the gut and induce inflammation [47]. The pathological accumulation of Aβ plaques is the key factor driving the inflammatory response of AD nerves, and the activation of microglia and astrocytes is the main source of proinflammatory cytokines (TNF-α, IL-1β, and IL-6) [33]. In this study, the deposition of Aβ plaques in the hippocampus of APP/PS1 rats was assessed via IHC after administration of intestinal dominant microbiota and *Enterococcus* (DM9112). The results showed that both treatments could significantly reduce the deposition area and number of Aβ plaques in the hippocampus. This experimental result is consistent with the conclusion of Wang et al. [48], who suggested that probiotic treatment can significantly reduce the deposition of Aβ amyloid plaques, which appears to be achieved by reducing oxidative stress. Oxidative stress may be associated with cognitive dysfunction through impaired signaling pathways and neuronal death, leading to plaque accumulation and neuroinflammation [49]. The results of qRT-PCR showed that the mRNA expression levels of APP and BACE1 in the brain tissue of APP/PS1 mice were significantly downregulated after administration of intestinal dominant microbiota and *Enterococcus* (DM9112), and the effect of intestinal dominant microbiota was more significant. The same trend was observed in terms of protein expression levels. The mRNA expression level and protein expression of IL-6 and TNF-α in the brain tissue of mice in both groups were significantly down-regulated, and the reperfusion effect of the dominant intestinal microbiota was more significant. *Lactobacillus* and *Bifidobacterium* can produce short-chain fatty acids, including butyric acid and propionic acid, which can act as anti-inflammatory agents and inhibit the activation of NF-KB by binding to G-protein-coupled receptors 43/41 while reducing the release of pro-inflammatory cytokines in immune-active microglial cells of the central nervous system [50,51]. Some studies have shown that systemic inflammatory states may exacerbate the progression of AD. Proinflammatory cytokines appear to be increased in the plasma and cerebrospinal fluid of AD patients and people with mild cognitive impairment [52]. The continuous release of cytokines by microglia and astrocytes appears to be due to the continuous deposition of Aβ peptides in the extracellular space.

Previous studies have confirmed that disturbance of the intestinal microbiota in AD patients leads to increased permeability of the intestinal epithelial barrier, and some proinflammatory neurotoxins such as lipopolysaccharide (LPS) and TNF-α may reach the CNS through the vagus nerve or systemic circulation, promoting microglial cell activation and neuroinflammation [53]. However, we measured the concentrations of IL-1β and TNF-α in serum. Compared with those of WT mice, the concentrations of IL-1β and TNF-α in the serum of APP/PS1 mice showed a decreasing trend, but there was no significant difference between the two treatment groups and the APP/PS1 group, which may be due to the small number of samples. The statistical results have a level of variance and are not representative.

In conclusion, we confirmed that the action of intestinal dominant microbiota on APP/PS1 mice can restore the richness and composition of intestinal microbiota, maintain the balance of intestinal microecology, effectively improve neuroinflammation, and reduce pathological deposition of Aβ plaques. In addition, the therapeutic effect of intestinal dominant microbiota was superior to that of a single probiotic and would be effective in addressing the existing problems of FMT therapy, thus representing a new option for the treatment of AD.

## Figures and Tables

**Table 1:** Intestinal dominant microbiota back to instillation bacteria scale.

Bacteria	Refill Volume of Bacteria (pcs)	Bacteria Enrichment Time during Live Bacteria Counting (h)	OD Value at Live Bacteria Count	Active Bacteria Count Results (CFU/mL)	Amount of Bacteria per OD at Live Bacteria Count (CFU/mL)
*Lactobacillus reuteri*	2 × 10^9^	24	1.34	1.87 × 10^8^	1.41 × 10^8^
*Bifidobacterium animalis*	2 × 10^9^	48	0.70	1.80 × 10^8^	2.57 × 10^8^
*Bacteroides ovatus*	1 × 10^9^	48	0.77	5.80 × 10^8^	7.53 × 10^8^
*Enterococcus faecium* (DM9112)	2 × 10^8^	24	0.54	5.10 × 10^8^	9.40 × 10^8^
*Streptococcus parasanguinis*	2 × 10^8^	24	0.69	0.70 × 10^8^	1.00 × 10^8^
*Staphylococcus nepalensis*	2 × 10^7^	24	0.14	0.60 × 10^8^	4.30 × 10^8^
*Fusobacterium gastrosuis*	2 × 10^6^	48	0.31	1.83 × 10^8^	5.85 × 10^8^
*Escherichia coli*	2 × 10^6^	24	0.30	3.30 × 10^8^	11.00 × 10^8^

## Data Availability

The datasets generated and analyzed during the current study are available in the [SRA] repository, (https://www.ncbi.nlm.nih.gov/search/all/?term=PRJNA947771, accessed on 10 July 2023).

## Supplementary Materials

The following supporting information can be downloaded at: https://www.mdpi.com/article/10.3390/microorganisms11122854/s1, Figure S1: Flow chart of the acquisition of dominant intestinal microbiota (*Lactobacillus reuteri*, *Enterococcus faecium*, *Escherichia coli*, *Streptococcus parasanguinis*, *Staphylococcus nepalensis*, *Bifidobacterium animalis*, *Bacteroides ovatus* and *Fusobacterium gastrosuis*); Figure S2: Colony morphology of intestinal dominant microbiota and microscopic morphology after Gram staining. (A,B) *Lactobacillus reuteri*. (C,D) *Bacteroides ovatus*. (E,F) *Bifidobacterium animalis*. (G,H) *Enterococcus faecium*. (I,J) *Streptococcus parasanguinis.* (K,L) *Escherichia coli.* (M,N) *Fusobacterium gastrosuis.* (O,P) *Staphylococcus nepalensis*; Figure S3: Technology Roadmap; Figure S4: PCR-DGGE imaging at different intervention periods. Different bands represent different microorganisms, including richness, Shannon index, cluster analysis diagram and principal component analysis diagram. (a) The first weekend of treatment. (b) The third weekend of treatment. (c) The fourth weekend of treatment; Figure S5: Changes in the intestinal microbiota of mice treated with *Enterococcus* (DM9112) and dominant intestinal microbiota by gavage for 5 weeks. (a) NMDS analysis. (b) Genus-level community composition heatmap; Table S1: Results of 16S rDNA sequencing of the dominant intestinal microbiota in NCBI comparison; Table S2: *Enterococcus* selective medium formulation; Table S3: Experimental grouping and sample size; Table S4: qRT-PCR primer sequences.
