# Supplementary material for: Application of Dominant Gut Microbiota Promises to Replace Fecal Microbiota Transplantation as a New Treatment for Alzheimer’s Disease
Source: Microorganisms. 2023 Nov 24;11(12):2854. doi: 10.3390/microorganisms11122854 (PMC10745325; doi:10.3390/microorganisms11122854)

Fig.S1. Flow chart of the acquisition of dominant intestinal microbiota (*Lactobacillus reuteri*, *Enterococcus faecium*, *Escherichia coli*, *Streptococcus parasanguinis*, *Staphylococcus nepalensis*, *Bifidobacterium animalis*, *Bacteroides ovatus* and *Fusobacterium gastrosuis*)

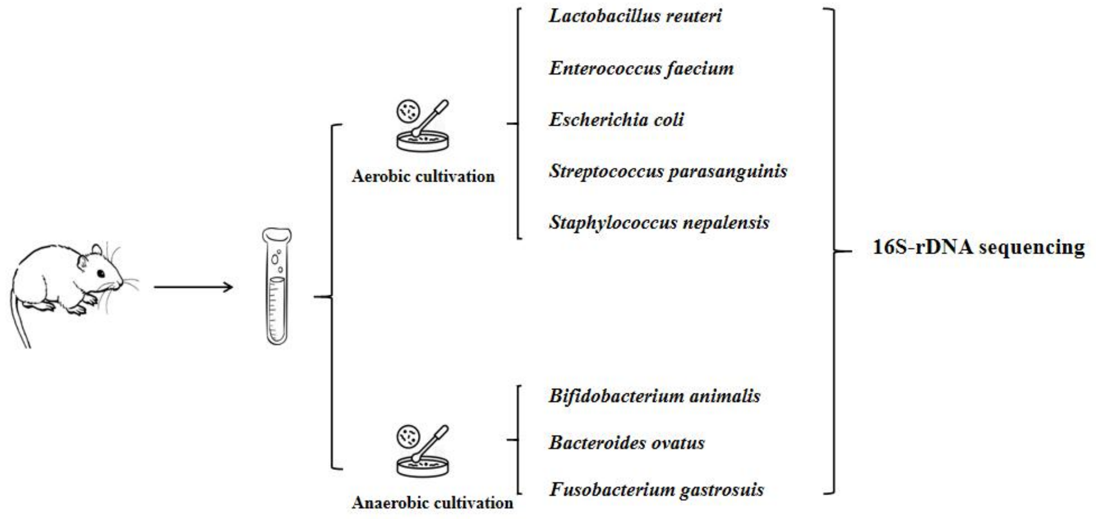

Supplement: Supplementary file 1 [file microorganisms-11-02854-s001.zip › PDF/Fig.S1.pdf]
