# Supplementary material for: Application of Dominant Gut Microbiota Promises to Replace Fecal Microbiota Transplantation as a New Treatment for Alzheimer’s Disease
Source: Microorganisms. 2023 Nov 24;11(12):2854. doi: 10.3390/microorganisms11122854 (PMC10745325; doi:10.3390/microorganisms11122854)

Fig.S2. Colony morphology of intestinal dominant microbiota and microscopic morphology after Gram staining. (A,B) *Lactobacillus reuteri*, (C,D) *Bacteroides ovatus*, (E,F) *Bifidobacterium animalis*, (G,H) *Enterococcus faecium*, (I,J) *Streptococcus parasanguinis*, (K,L) *Escherichia coli*, (M,N) *Fusobacterium gastrosuis*, (O,P) *Staphylococcus nepalensis*.

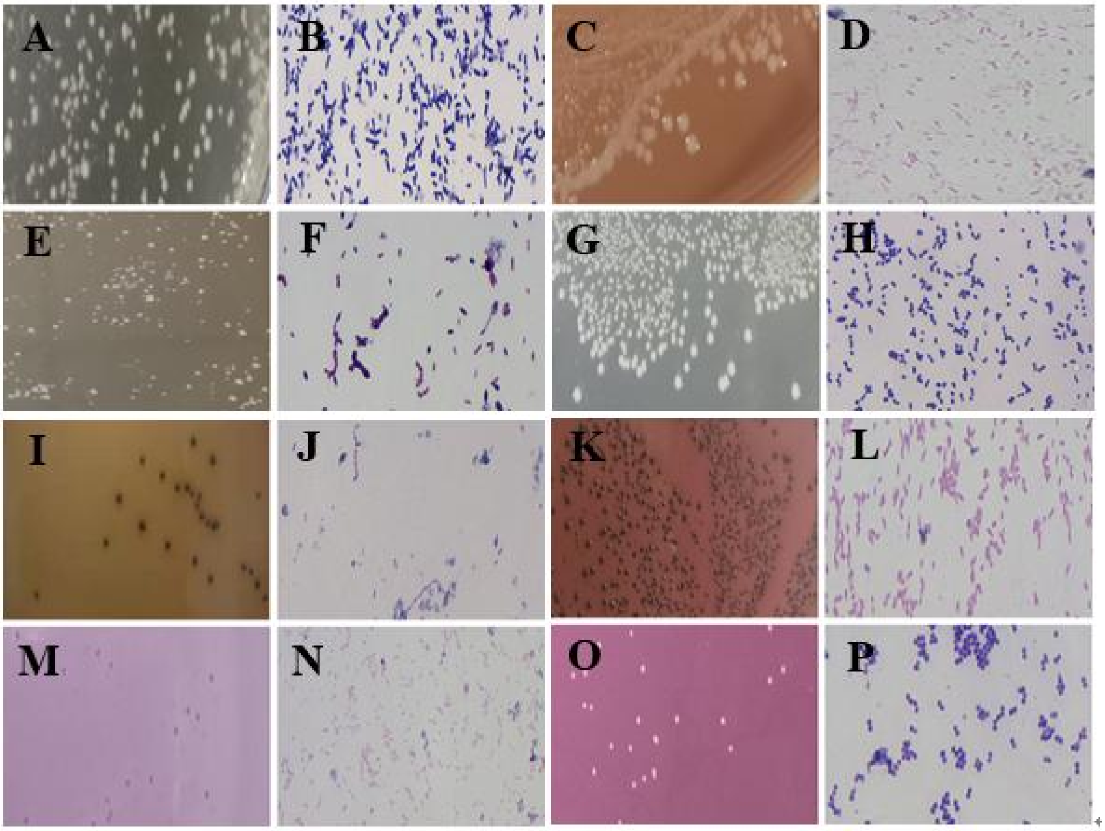

Supplement: Supplementary file 1 [file microorganisms-11-02854-s001.zip › PDF/Fig.S2.pdf]
