# Supplementary material for: Application of Dominant Gut Microbiota Promises to Replace Fecal Microbiota Transplantation as a New Treatment for Alzheimer’s Disease
Source: Microorganisms. 2023 Nov 24;11(12):2854. doi: 10.3390/microorganisms11122854 (PMC10745325; doi:10.3390/microorganisms11122854)
Supplement: Supplementary file 1 [file microorganisms-11-02854-s001.zip › PDF/Fig.S4.pdf]

Fig.S4. PCR-DGGE imaging at different intervention periods. Different bands represent different microorganisms, including richness, Shannon index, cluster analysis diagram and principal component analysis diagram. (a) The first weekend of treatment. (b) The third weekend of treatment. (c) The fourth weekend of treatment. Values represent the mean ± S.E.M. ＊P＜0.05， ＊＊P＜0.01. n=3mice/group.

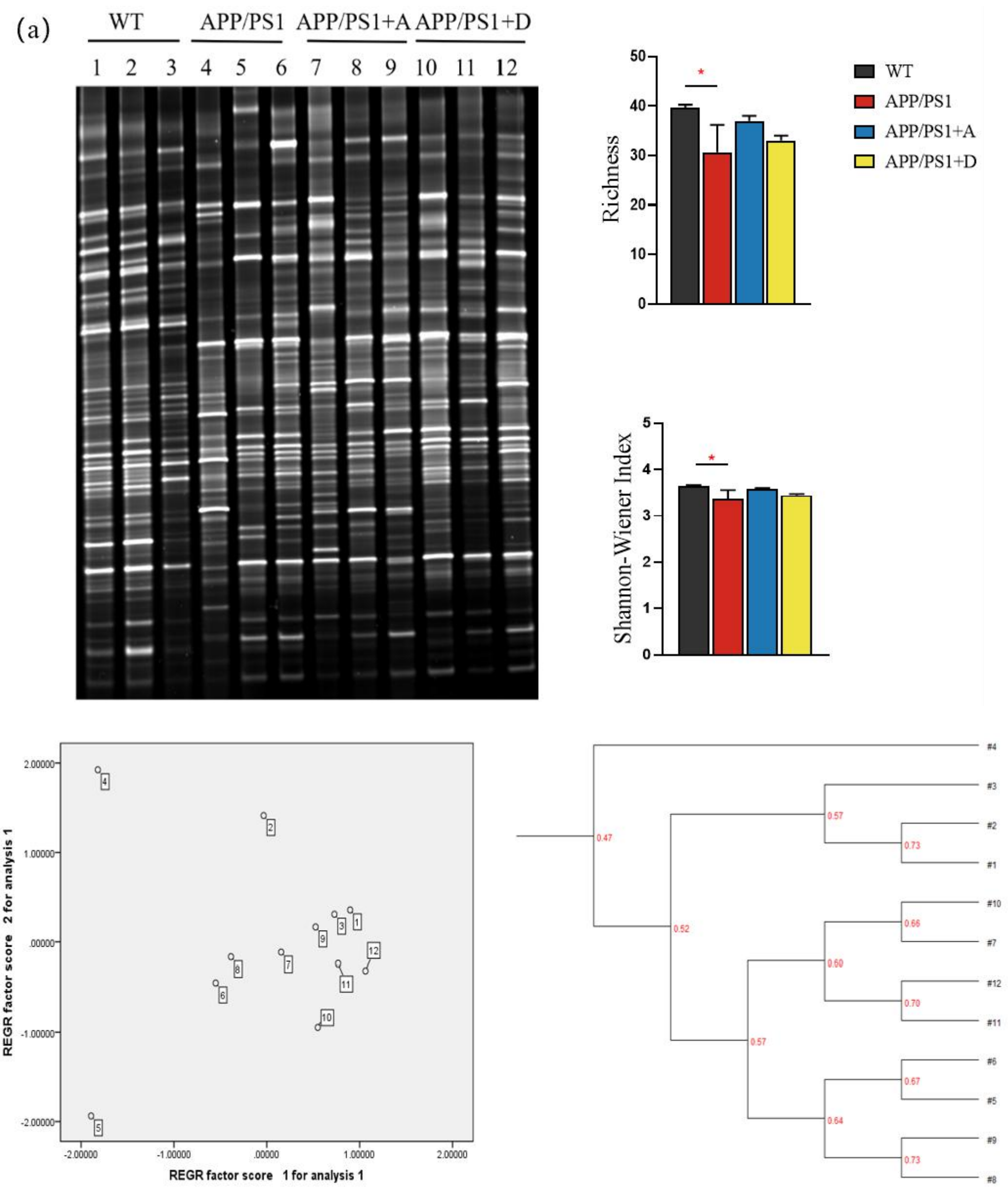

(b)

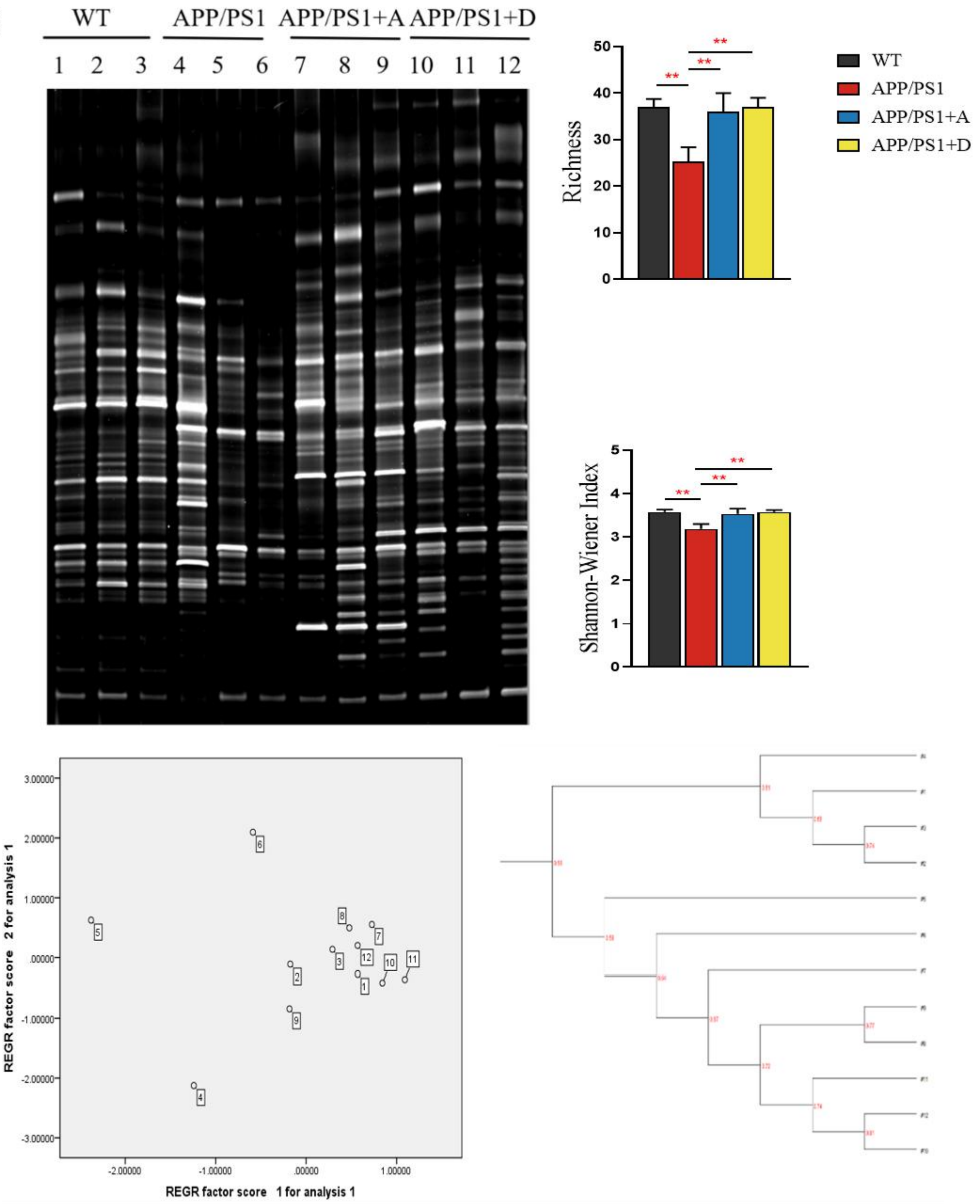

WT
APP/PS1
APP/PS1+A
APP/PS1+D
1 2 3 4 5 6 7 8 9 10 11 12
Richness
50
40
30
20
10
0
**
Shannon-Wiener Index
5
4
3
2
1
0
REGR factor score 2 for analysis 1
REGR factor score 1 for analysis 1
3.00000
2.00000
1.00000
.00000
-1.00000
-2.00000
-3.00000
-2.00000
-1.00000
.00000
1.00000

(c)

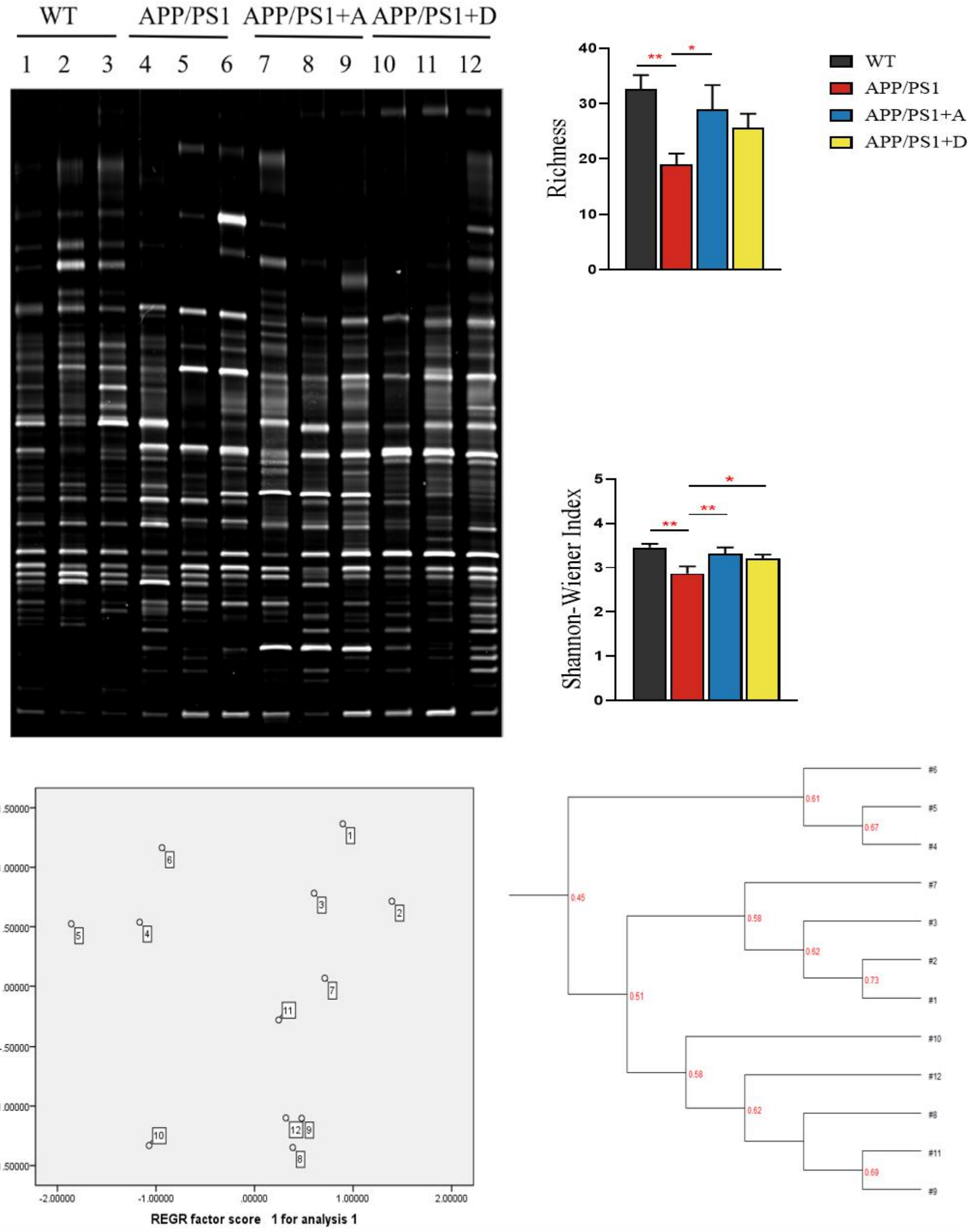

WT
APP/PS1
APP/PS1+A
APP/PS1+D
1 2 3 4 5 6 7 8 9 10 11 12
Richness
Shannon-Wiener Index
REGR factor score 1 for analysis 1
REGR factor score 2 for analysis 1
