# Supplementary material for: Application of Dominant Gut Microbiota Promises to Replace Fecal Microbiota Transplantation as a New Treatment for Alzheimer’s Disease
Source: Microorganisms. 2023 Nov 24;11(12):2854. doi: 10.3390/microorganisms11122854 (PMC10745325; doi:10.3390/microorganisms11122854)

Fig.S5. Changes in the intestinal microbiota of mice treated with *Enterococcus* (DM9112) and dominant intestinal microbiota by gavage for five weeks. (a) NMDS analysis. (b) Genus-level community composition heatmap. Values represent the mean ± S.E.M. *n*=6mice/group.

(a)

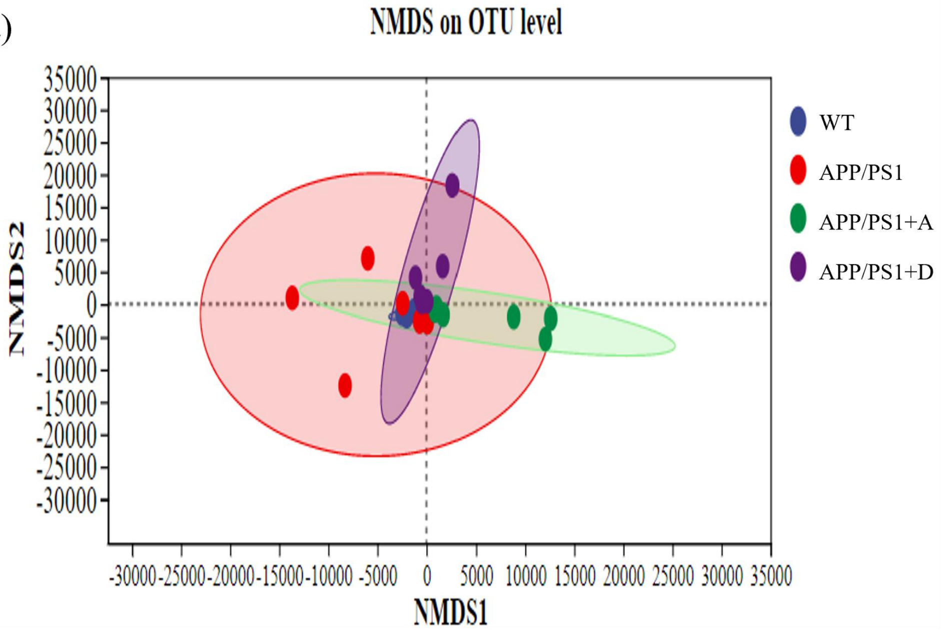

(b)

Community heatmap analysis on Genus level

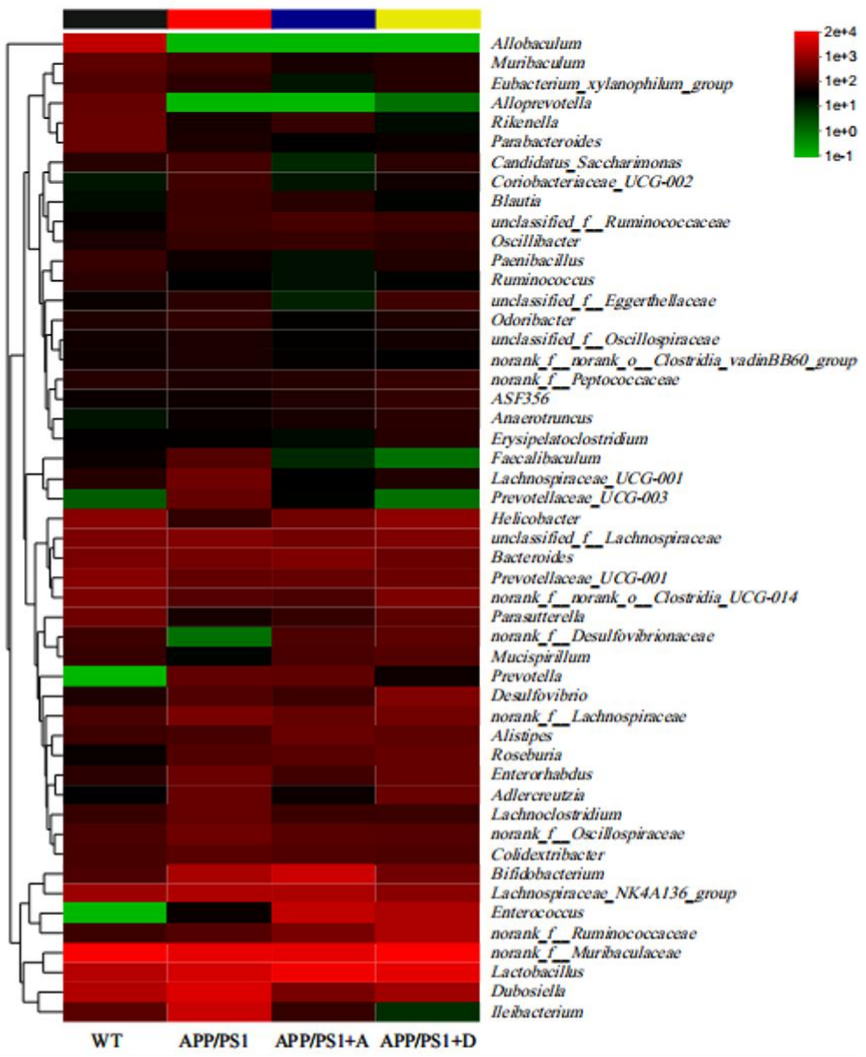

Supplement: Supplementary file 1 [file microorganisms-11-02854-s001.zip › PDF/Fig.S5.pdf]
