# Supplementary material for: Application of Dominant Gut Microbiota Promises to Replace Fecal Microbiota Transplantation as a New Treatment for Alzheimer’s Disease
Source: Microorganisms. 2023 Nov 24;11(12):2854. doi: 10.3390/microorganisms11122854 (PMC10745325; doi:10.3390/microorganisms11122854)
Supplement: Supplementary file 1 [file microorganisms-11-02854-s001.zip › PDF/Table S1.pdf]

Table S1. Results of 16S rDNA sequencing of the dominant intestinal microbiota in NCBI comparison

**Description**	**Max Score**	**Total Score**	**Query Cover**	**E value**	**Per.Ident**	**Accession**
Lactobacillus reuteri	2719	2719	99%	0	99.53%	NR_075036.1
Bifidobacterium animalis	1724	1724	99%	0	98.96%	KR_364744.1
Bacteroides ovatus	891	891	98%	0	99.59%	KP_944116.1
Streptococcus parasanguinis	896	896	98%	0	99%	MT_544746.1
Enterococcus faecium	2652	2652	97%	0	99.59%	NR_115764.1
Escherichia coli	2582	2582	99%	0	99.51%	NR_114042.1
Staphylococcus nepalensis	2593	2593	99%	0	99.37%	NR_028996.1
Fusobacterium gastrosuis	2401	2401	100%	0	98.12%	NR_146837.2
