# Supplementary material for: Application of Dominant Gut Microbiota Promises to Replace Fecal Microbiota Transplantation as a New Treatment for Alzheimer’s Disease
Source: Microorganisms. 2023 Nov 24;11(12):2854. doi: 10.3390/microorganisms11122854 (PMC10745325; doi:10.3390/microorganisms11122854)
Supplement: Supplementary file 1 [file microorganisms-11-02854-s001.zip › PDF/Table S2.pdf]

Table S2. *Enterococcus* selective medium formulation

**Ingredient**	**Dose**
Tomato juice	200mL
Peptone	15g
Yeast extract	6g
Glucose	20g
Sodium chloride	5g
Tween 80	1mL
Soluble starch	0.5g
Agar	15g

Add HCl to PH=6.7±0.2, heat and dissolve in 1000 mL deionized water, and autoclave at 116 ℃ for 30 min.
