# Supplementary material for: Application of Dominant Gut Microbiota Promises to Replace Fecal Microbiota Transplantation as a New Treatment for Alzheimer’s Disease
Source: Microorganisms. 2023 Nov 24;11(12):2854. doi: 10.3390/microorganisms11122854 (PMC10745325; doi:10.3390/microorganisms11122854)
Supplement: Supplementary file 1 [file microorganisms-11-02854-s001.zip › PDF/Table S3.pdf]

Table S3. Experimental grouping and sample size

Experimental groups	DGGE	16S rRNA	IHC	qRT-PCR	ELISA	Western blot	Death
WT	6	6	3	4	4	3	7
APP/PS1	6	6	3	4	3	3	6
APP/PS1+A	6	6	3	4	4	3	7
APP/PS1+D	6	6	3	4	4	3	7
