# Supplementary material for: Application of Dominant Gut Microbiota Promises to Replace Fecal Microbiota Transplantation as a New Treatment for Alzheimer’s Disease
Source: Microorganisms. 2023 Nov 24;11(12):2854. doi: 10.3390/microorganisms11122854 (PMC10745325; doi:10.3390/microorganisms11122854)
Supplement: Supplementary file 1 [file microorganisms-11-02854-s001.zip › PDF/Table S4.pdf]

Table S4. qRT-PCR primer sequences

GENES	Forward primers	Reverse primers
GAPDH	5’-TGTGTCCGTCGTCGTGGATCTGA-3’	5’-TTGCTGTTGAAGTCGCAGGAG-3’
APP	5’-TGAACAAGCCGAGACCG-3’	5’-TCGTGGGAAGTTTATCAGG-3’
BACE1	5’-CCACAGACGCTCAACATCC-3’	5’-AGGTCGGTGCCCAGTTC-3’
IL-6	5’-TTGCCTTCTTGGGACTGAT-3’	5’-TTGCCATTGCACAACTCTT-3’
IL-1β	5’-TTGCCTTCTTGGGACTGAT-3’	5’-TTGCCTTCTTGGGACTGAT-3’
