# Supplementary figures and images for: Application of Dominant Gut Microbiota Promises to Replace Fecal Microbiota Transplantation as a New Treatment for Alzheimer’s Disease
Source: Microorganisms. 2023 Nov 24;11(12):2854. doi: 10.3390/microorganisms11122854 (PMC10745325; doi:10.3390/microorganisms11122854)

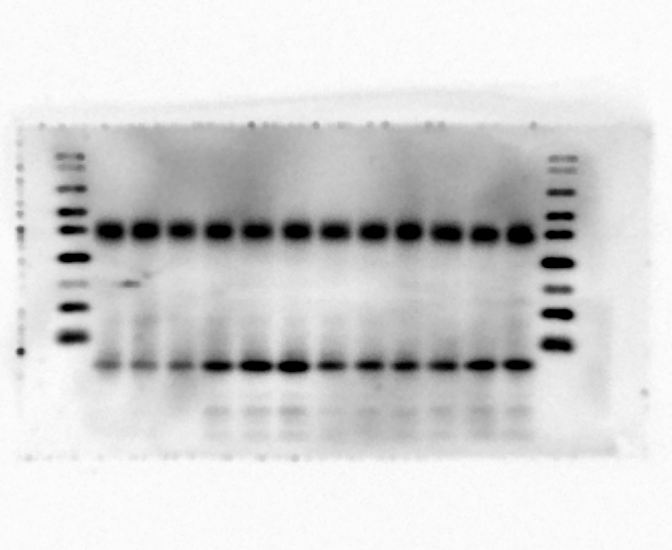

Supplement: Supplementary file 1 [file microorganisms-11-02854-s001.zip › Original Images for Blots and Gels/Western blot/Figure 6e.tif]

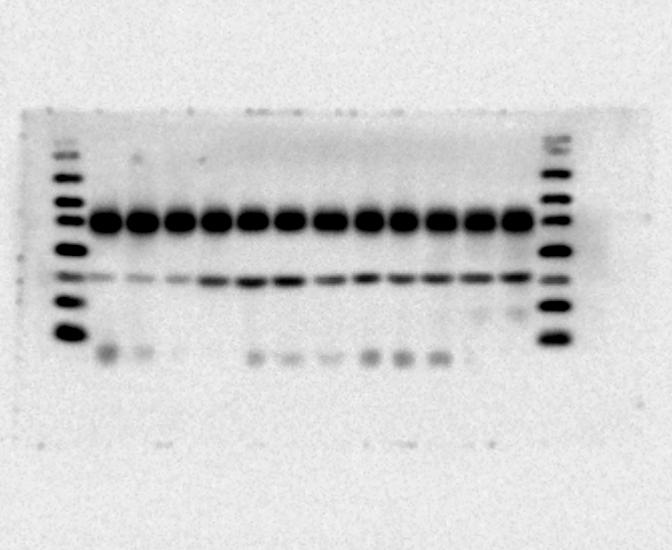

Supplement: Supplementary file 1 [file microorganisms-11-02854-s001.zip › Original Images for Blots and Gels/Western blot/Figure 6f.tif]

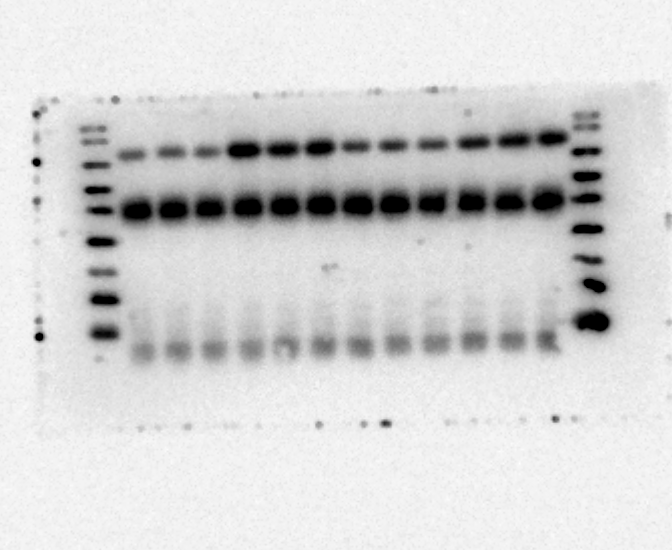

Supplement: Supplementary file 1 [file microorganisms-11-02854-s001.zip › Original Images for Blots and Gels/Western blot/Figure 6g.tif]

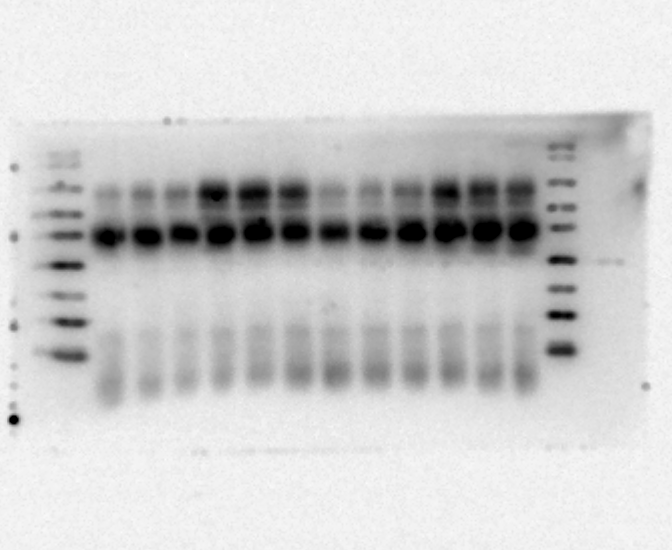

Supplement: Supplementary file 1 [file microorganisms-11-02854-s001.zip › Original Images for Blots and Gels/Western blot/Figure 6h.tif]

Fig.S3. Technology Roadmap

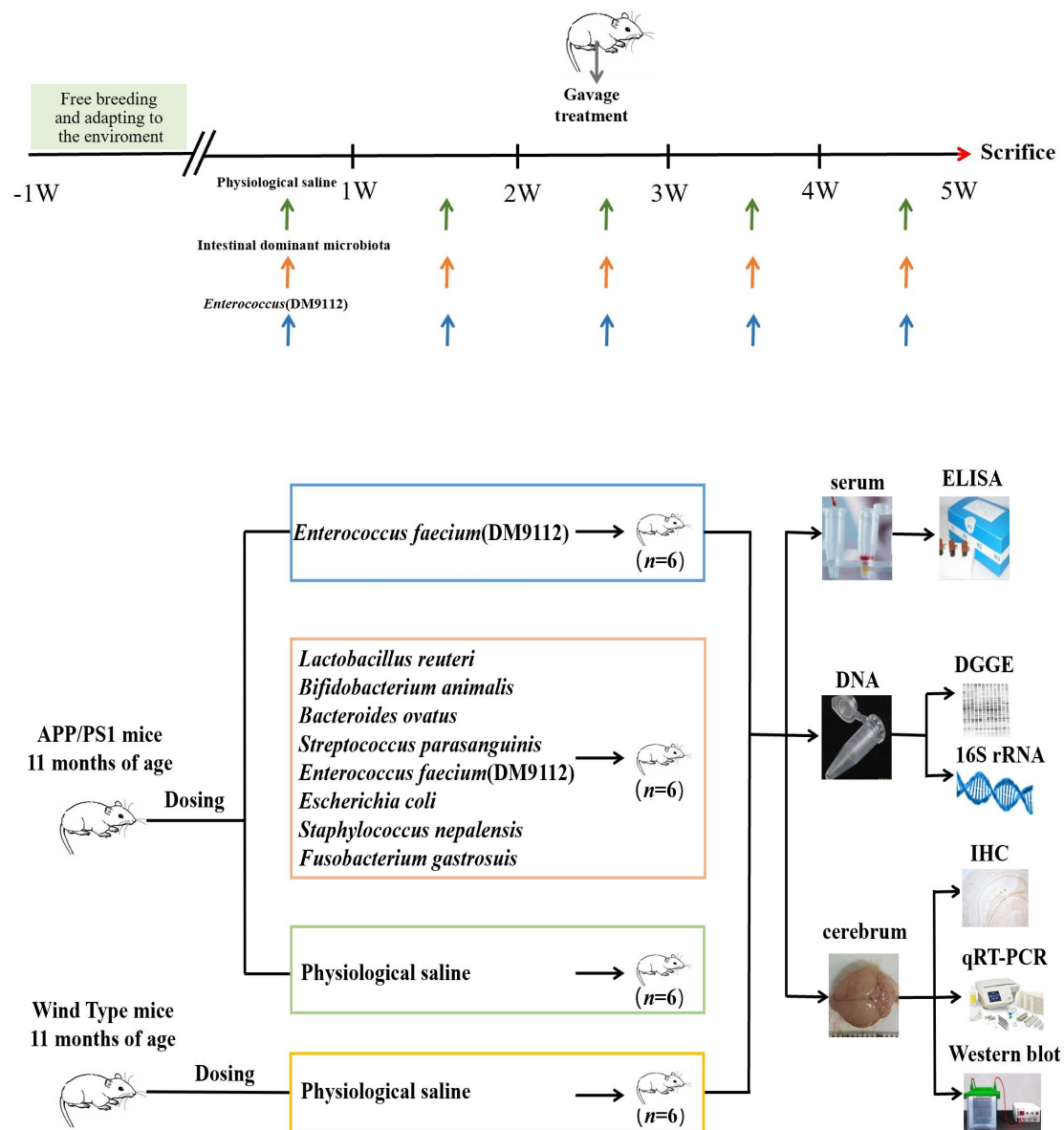

Supplement: Supplementary file 1 [file microorganisms-11-02854-s001.zip › PDF/Fig.S3.pdf]
